# Betaine regulates the gut-liver axis: a therapeutic approach for chronic liver diseases

**DOI:** 10.3389/fnut.2025.1478542

**Published:** 2025-03-24

**Authors:** Sathish Kumar Perumal, Madan Kumar Arumugam, Natalia A. Osna, Karuna Rasineni, Kusum K. Kharbanda

**Affiliations:** ^1^Research Service, Department of Veterans Affairs, Nebraska-Western Iowa Health Care System, Omaha, NE, United States; ^2^Department of Internal Medicine, University of Nebraska Medical Center, Omaha, NE, United States; ^3^Cancer Biology Lab, Centre for Molecular and Nanomedical Sciences, Sathyabama Institute of Science and Technology, Chennai, Tamil Nadu, India; ^4^Department of Pharmacology and Experimental Neuroscience, University of Nebraska Medical Center, Omaha, NE, United States; ^5^Department of Biochemistry & Molecular Biology, University of Nebraska Medical Center, Omaha, NE, United States

**Keywords:** betaine, liver, alcohol-associated liver disease, metabolic dysfunction-associated steatotic liver disease, gut-liver axis, intestinal barrier integrity, gut microbiome

## Abstract

Chronic liver disease is defined by persistent harm to the liver that might result in decreased liver function. The two prevalent chronic liver diseases are alcohol-associated liver disease (ALD) and metabolic dysfunction-associated steatotic liver disease (MASLD). There is ample evidence that the pathogenesis of these two chronic liver diseases is closely linked to gastrointestinal dysfunctions that alters the gut-liver crosstalk. These alterations are mediated through the imbalances in the gut microbiota composition/function that combined with disruption in the gut barrier integrity allows for harmful gut microbes and their toxins to enter the portal circulation and reach the liver to elicit an inflammatory response. This leads to further recruitment of systemic inflammatory cells, such as neutrophils, T-cells, and monocytes into the liver, which perpetuate additional inflammation and the development of progressive liver damage. Many therapeutic modalities, currently used to prevent, attenuate, or treat chronic liver diseases are aimed at modulating gut dysbiosis and improving intestinal barrier function. Betaine is a choline-derived metabolite and a methyl group donor with antioxidant, anti-inflammatory and osmoprotectant properties. Studies have shown that low betaine levels are associated with higher levels of organ damage. There have been several publications demonstrating the role of betaine supplementation in preventing the development of ALD and MASLD. This review explores the protective effects of betaine through its role as a methyl donor and its capacity to regulate the protective gut microbiota and maintain intestinal barrier integrity to prevent the development of these chronic liver diseases. Further studies are needed to enhance our understanding of its therapeutic potential that could pave the way for targeted interventions in the management of not only chronic liver diseases, but other inflammatory bowel diseases or systemic inflammatory conditions.

## Introduction

A variety of illnesses are grouped together under the umbrella term “chronic liver disease,” which is defined by persistent harm to the liver that might result in decreased liver function (1–3). Common causes for liver diseases are hepatitis B and C infections, autoimmune illnesses, hereditary factors and Western lifestyle, which includes dietary changes, inactivity, and alcohol intake (4–8). Hippocrates, the father of modern medicine claimed ~2,500 years ago that “all diseases begin in the gut.” There is now ample evidence that indeed gut dysfunction is involved in the pathogenesis of many diseases especially those of the liver (9–13). This is because the gut and liver are anatomically linked via the portal system (14). Normally, the gut epithelial cells maintain barrier integrity through microvilli, tight junctions, and production of antimicrobial peptides (15, 16). These gastrointestinal epithelial cell boundary frameworks prevent the translocation of most of gut luminal content and in healthy conditions only small number of microbial products can reach the liver (17). In this mutual relationship, the liver acts as a second firewall toward the low number of potentially harmful substances translocated from the gut that are eliminated by the liver resident macrophages, Kupffer cells (14). However, alterations in gut homeostasis characterized by changes in the composition of the gut microbiota, microbial products, antimicrobial peptide levels and mucosal barrier disruption play a major role in the onset and progression of liver diseases including two prevalent diseases, alcohol-associated liver disease (ALD) and metabolic dysfunction-associated steatotic liver disease (MASLD) (9–11, 13, 18, 19).

Betaine, a naturally occurring derivative of choline, plays an important role in various cellular processes due to its function as a methyl donor in the methionine-homocysteine cycle (20). Studies show that the role of betaine in remethylating homocysteine to methionine through the betaine-homocysteine methyltransferase (BHMT)-catalyzed reaction helps reduce homocysteine and S-adenosylhomocysteine (SAH) levels (21–23), factors linked to adipose, gut and liver dysfunction (24–28). In the context of liver diseases, betaine has been shown to mitigate lipid accumulation, oxidative stress, proteasomal/lysosomal dysfunction, hepatocyte death, and inflammation, making it a promising therapeutic agent for ALD (24, 29, 30) and MASLD (31, 32). Additionally, betaine is significant for gut health as it stabilizes intestinal barrier integrity, regulates osmolality, and modulates the gut microbiota (33, 34). In addition, betaine increases the villus height and crypt depth and by maintaining the tight junctional protein complexes prevents luminal antigens/microbial translocation from the gut to the liver (35). These effects on the gut-liver axis highlight its therapeutic potential in preventing liver inflammation and progressive injury. Hence, in this review, we highlight the protective role of betaine on the gut-liver axis and its relevance in managing liver diseases.

## Alcohol-associated liver disease (ALD)

Alcohol-associated liver disease is liver damage that arises from excessive consumption of beverages that contain ethyl alcohol (ethanol) (36–38). As the principal site of alcohol absorption, the gastrointestinal tract plays an important role in regulating the systemic availability of ethanol (39, 40). While ethanol metabolism in the gastrointestinal tract locally generates low levels of acetaldehyde and other toxic metabolites, the liver is the primary organ that carries out ethanol metabolism and consequently sustains the greatest organ damage from heavy drinking (41, 42). The hepatic metabolism of ethanol occurs through both oxidative and non-oxidative pathways. Oxidative ethanol metabolism is catalyzed by alcohol dehydrogenase (ADH) which converts ethanol to acetaldehyde, which is subsequently oxidized to acetate (43, 44). In addition, another major ethanol-metabolizing enzyme is cytochrome P450 2E1 (CYP2E1), a microsomal enzyme that is significantly induced by ethanol consumption (45).

**Figure 1:**
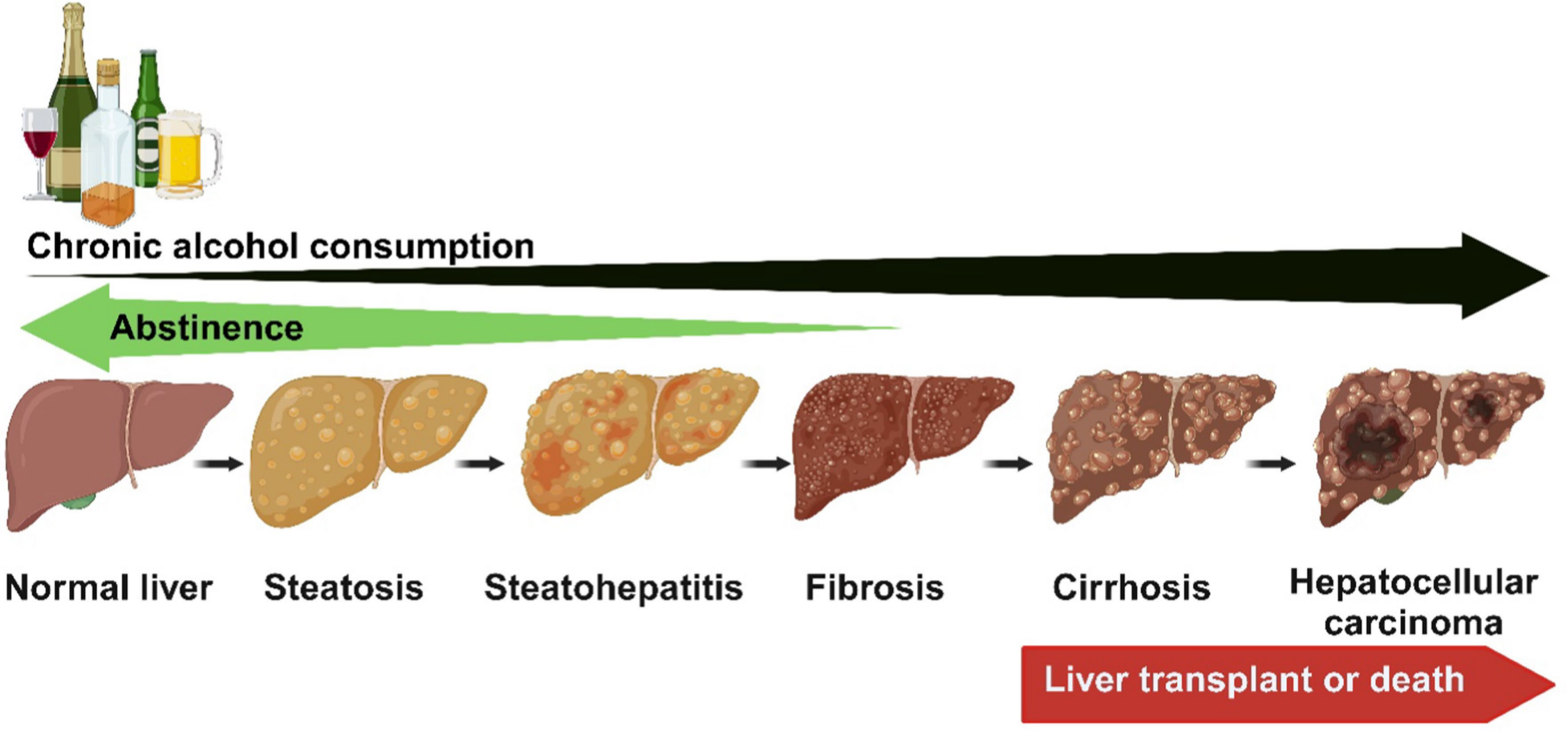
The spectrum of ALD encompasses a broad spectrum of pathologies, including steatosis (fatty liver), which can progress to steatohepatitis (steatosis with inflammation and necrosis) and fibrosis. If harmful drinking continues, liver cirrhosis can develop, during which hepatocytes dedifferentiate, lose their phenotype, and can become neoplastic, resulting in hepatocellular carcinoma. The early stages of ALD reverse with abstinence. Created with biorender.com.

Alcohol-associated liver disease encompasses a broad spectrum of pathologies, including steatosis (fatty liver), which can progress to steatohepatitis [steatosis with inflammation and necrosis and fibrosis (Figure 1)]. If harmful drinking continues, liver cirrhosis can develop, during which hepatocytes dedifferentiate, lose their phenotype, and can become neoplastic, resulting in hepatocellular carcinoma (46–48). ALD progression from steatosis to more severe liver damage is associated with the amount, type, pattern, and duration of alcohol consumption (45). Other factors involved in the pathogenesis and progression of ALD include detrimental changes in gut microbiota and intestinal barrier disruption (45, 49–51). This translocation of gut luminal pathobionts and products triggers inflammatory responses in the liver, involving resident hepatic macrophages, Kupffer cells, and other infiltrating immune cells that activates hepatic stellate cells (HSC), causing aberrant production of extracellular matrix (ECM) proteins and fibrosis development. Furthermore, due to aberrant ECM production by activated HSCs, liver sinusoidal endothelial cells undergo capillarization, including the loss of fenestrations and a shift toward a vascular phenotype. These changes collectively contribute to the development of advanced alcohol-induced liver injury (52, 53).

## Metabolic dysfunction-associated steatotic liver disease (MASLD)

Metabolic dysfunction-associated steatotic liver disease is an important hepatic manifestation of metabolic syndrome (54) characterized by fat accumulation in more than 5% of hepatocytes (55). MASLD is the most widespread chronic liver disease, impacting around one-quarter of the global population due to the growing rates of metabolic syndrome, obesity, and diabetes (56, 57). Patients with MASLD typically follow an unhealthy dietary pattern marked by elevated intake of saturated fats, cholesterol, and fructose and low physical activity (54). Conversely, there is a lower consumption of antioxidant vitamins and polyunsaturated fats (58). MASLD displays a similar spectrum of progression as ALD ranging from steatosis through steatohepatitis, hepatitis, cirrhosis, and to hepatocellular carcinoma (54, 59–61). The early stages of MASLD, as ALD, are marked by steatosis that progresses to metabolic dysfunction-associated steatohepatitis (MASH) distinguished by hepatocellular damage that includes hepatocyte ballooning degeneration, diffuse lobular inflammation, and fibrosis (62). MASH is increasingly being recognized as the leading indication for liver transplant listing for women and is expected to overtake ALD as the leading liver transplant indication for all patients within the next few years (60, 63).

**Figure 2:**
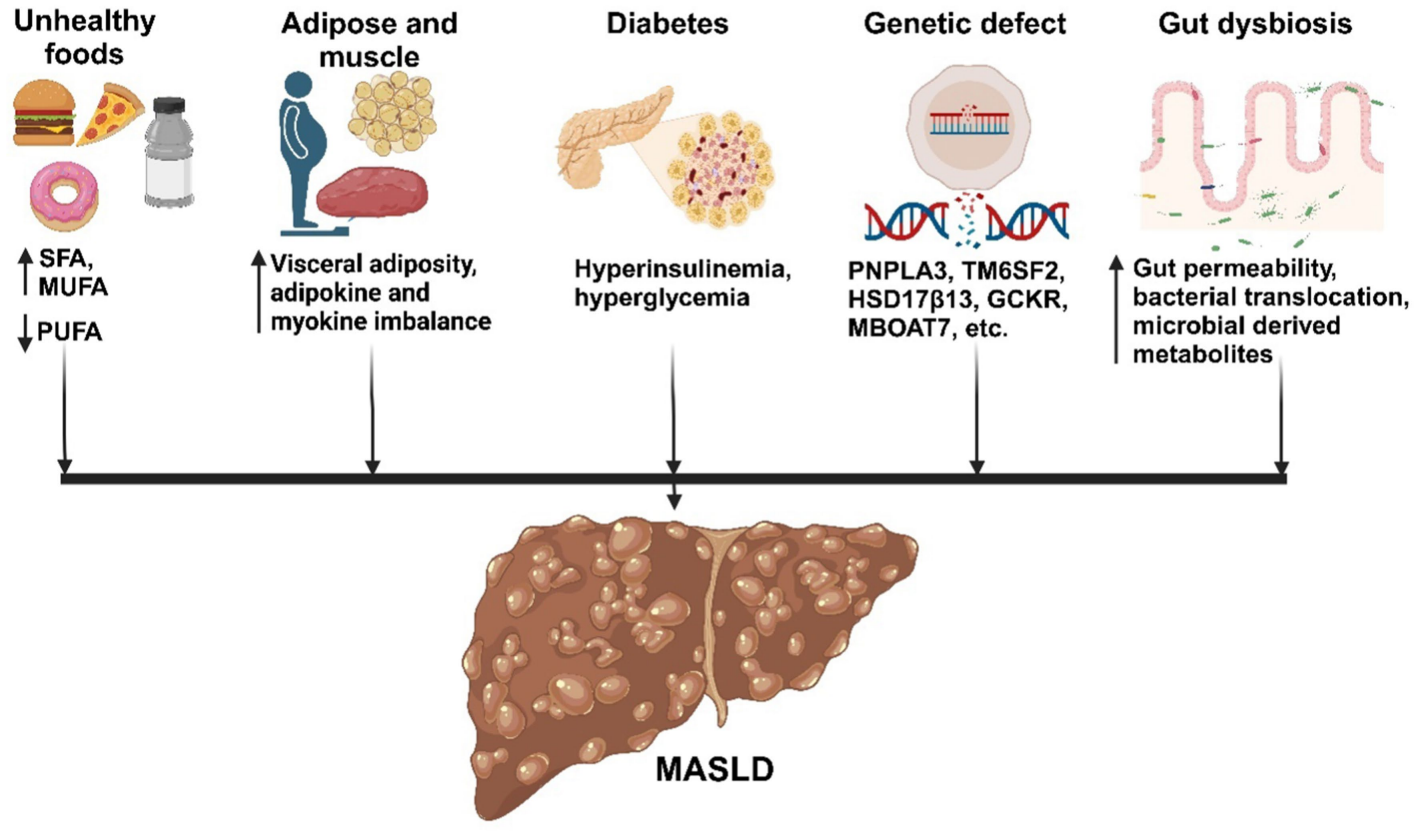
Factors influencing the development of MASLD. Metabolic factors promote the accumulation of fat in the liver, leading to inflammation, cell death, and fibrosis. Adipose tissue and muscle produce adipokines and myokines, respectively, which promote inflammation and oxidative stress in the liver. Additionally, the gut microbiota influences fat accumulation and inflammation in the liver, contributing to MASLD development. Created with biorender.com.

The underlying mechanism for the initiation and progression of MASLD is complex and multifactorial. Different theories have been postulated leading initially to the ‘two-hit’ hypothesis (64). According to this, the ‘first hit’ is induced by the accumulation of lipids in hepatocytes from high-caloric intake, obesity, insulin resistance, which makes the liver vulnerable to other damaging factors that are referred to as the ‘second hits’, such as oxidative stress, genetic polymorphisms (65). These second hits increase the vulnerability to the disease, activation of inflammatory pathways triggered by the release of pro-inflammatory cytokines by Kupffer cells or from adipocytes, dysregulated hepatocyte apoptosis, and activation of HSC, among others (66). When acting individually, these factors drive the progression from macrovesicular steatosis to MASH, which involves a gradual increase in liver injury parameters in association with hepatocyte apoptosis, inflammation, and fibrogenesis. Fibrosis advances from periportal to bridging fibrosis, eventually leading to cirrhotic remodeling, liver failure, and can ultimately result in hepatocellular carcinoma (67). The simplistic and outdated ‘two-hit’ hypothesis has been replaced by a “multi-hit’ model that integrates various interconnected processes, as shown in Figure 2, where multiple factors act synergistically, encompassing lipotoxicity, gut dysfunction, altered gut-liver axis and innate immune activation, within the context of genetic and environmental factors (68).

## Liver-gut axis

A continuous “dialogue” exists between the gut and liver, heavily influenced by the trillions of microbes residing within, the food we consume, and the world around us. This multifaceted communication has been named the gut-liver axis (69). In both the gut and liver, a “symphony of nutrients, microbial antigens, metabolites, and bile acids” conducts the “orchestra” of metabolism and immune responses. This intricate “dance,” in turn, shapes the composition and function of the gut microbiome. Gut microbes aren’t just passive residents; they are active players influencing our health throughout the body. Generally, gut and liver are in constant “conversation” for the digestion, nutrient processing, and even filtering out microbial leftovers. However, in experimental models of liver disease, this harmonious interplay is disrupted. A weakened intestinal barrier or a “leaky gut” allows for the melody to go off-key, that allows harmful gut microbes and their toxins to enter the portal circulation and reach the liver to elicit an inflammatory response by the Kupffer cells (70, 71). This local hepatic inflammation leads to further recruitment of systemic inflammatory cells, such as neutrophils, T-cells and monocytes, which perpetuate additional inflammation, hepatic fibrosis, hepatocyte cell death (68, 72, 73) that ultimately leads to rapid progression to multiple organ failure (74).

Indeed, alterations in the gut-liver axis characterized by increased portal and circulating levels of gut microbes/microbial components from compromised gut barrier and microbiota changes has emerged as central mediators in promoting ALD and MASLD progression (50, 51, 75–78).

### The gut barrier

Three main defense mechanisms make up the intestinal barrier function: (1) The immune barrier, which is made up of gut associated lymphoid tissue (GALT), effector and regulatory T cells, IgA-producing B cells, group 3 innate lymphoid cells, resident macrophages, and dendritic cells in the lamina propria; (2) the biological barrier, which is made up of normal intestinal flora, is responsible for colonization resistance; and (3) the mechanical barrier, which consists of intestinal epithelial cells that line the lumen (79, 80). This physical barrier is finely regulated by large molecular complexes which link intestinal epithelial cells to each other and seal the intercellular spaces on the luminal surface (81). These intercellular complexes regulate passage of molecules through the paracellular spaces (82) that simultaneously performs two opposing tasks. It selectively permits the passage of necessary nutrients from the intestinal lumen into the bloodstream and the internal milieu at large; on the other hand, it prevents the entry of hazardous substances such as microbes, the luminal antigens and proinflammatory factors (83). The most apical part of the junctional complexes are tight junctions (TJs), which are highly specialized and dynamic supramolecular entities composed of several transmembrane proteins (such as the claudin family, junctional adhesion molecule (JAM)-A, occludin, tricellulin) and the cytoplasmic plaques comprised of scaffolding proteins [zonulae occludens (ZO-1, ZO-2, ZO-3, cingulin)] and effector proteins (84–87) that provide a physical link to the cytoskeleton. There are also biochemical components, including enzymes and antimicrobial proteins that regulate the survival and proliferation of gut microbes (88, 89).

### Gut microbiota

**Figure 3:**
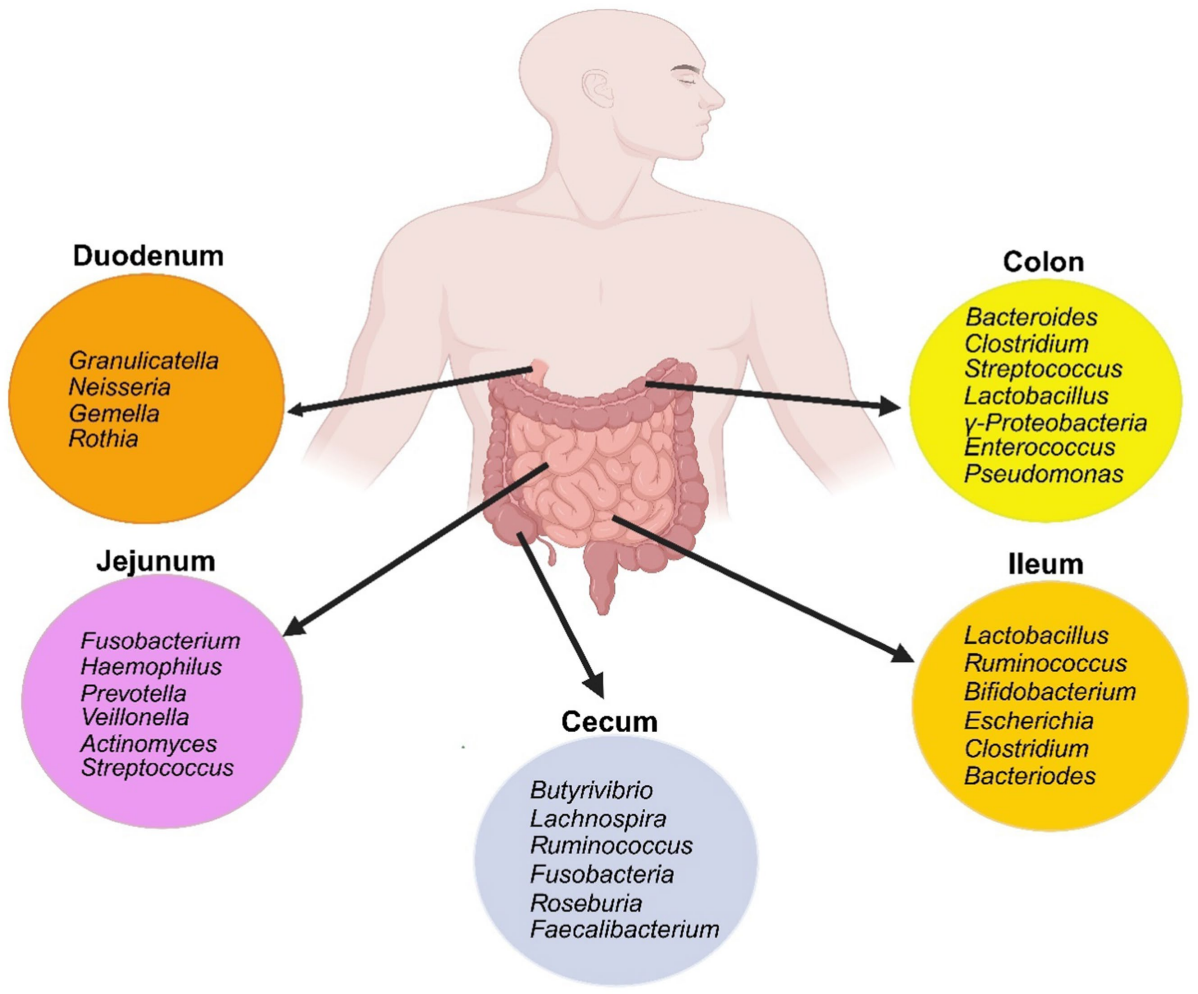
Schematic of the gut bacterial composition along the different segments of the small and large intestine. Created with biorender.com.

The gastrointestinal tract harbors a complex and dynamic population of microorganisms, known as the gut microbiota. The gut microbiome encompasses a complete array of microorganisms including bacteria, viruses, fungi, and protozoa (90). It is an intricate ecosystem consisting of 10–100 trillion microorganisms, with gut bacteria being the most well-studied component (88). The major gut bacterial species belong to the phyla Firmicutes and Bacteroidetes (Figure 3), while smaller proportions belong to Actinobacteria, Fusobacteria, Proteobacteria, and Verrucomicrobia (91).

These microorganisms contribute to various physiological functions, such as strengthening gut integrity, shaping the intestinal epithelium, harvesting energy, protecting against pathogens, and regulating host immunity (92). The microbiome has significantly enriched metabolism of glycans, amino acids, xenobiotics, methanogenesis and 2-methyl-d-erythritol 4-phosphate pathway-mediated biosynthesis of vitamins and isoprenoids (88, 93). The concentration and makeup of bacterial species vary throughout the entire gastrointestinal tract. These differences can vary between individuals due to age, ethnicity, lifestyle, medications, and dietary patterns indicating that the composition of the microbiota is shaped by both host and environmental selection pressures. The complexity of this symbiotic relationship and its implications for overall health are highlighted by the fact that the survival and proliferation of gut microbes are ultimately determined by their phenotypic features (94, 95). The intricate intestinal barrier comprised of physical, biochemical, and immunological elements acts as a shield, preventing the host immune system from being exposed to the microbiota while still permitting vital interactions to take place (96).

**Figure 4:**
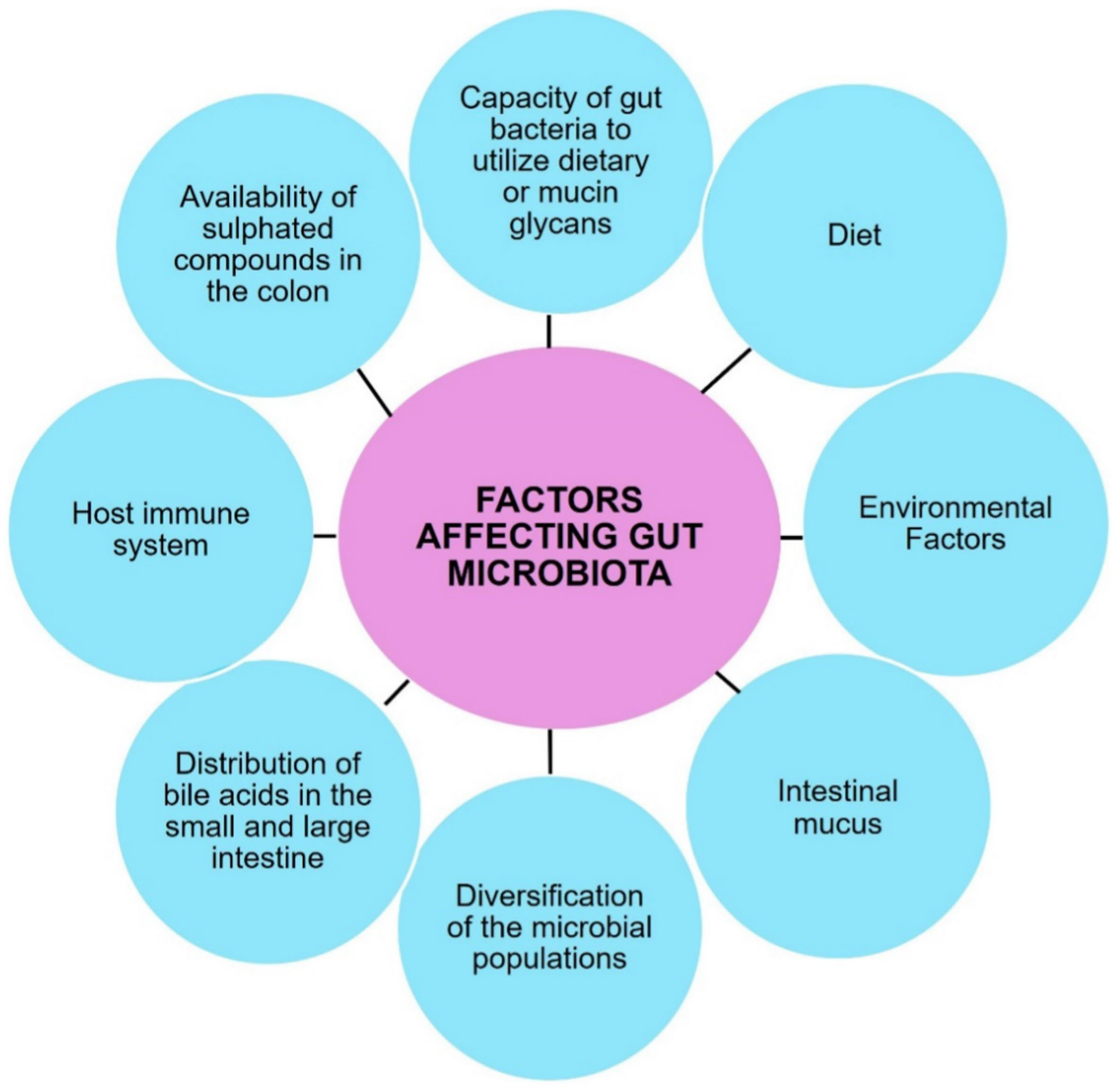
Various factors that regulate the gut microbiome.

In summary, the intricate interplay between the gut microbiota and the host’s gastrointestinal tract is essential for maintaining overall health and homeostasis as shown in Figure 4.

### Alterations in gut homeostasis and liver disease pathogenesis

**Figure 5:**
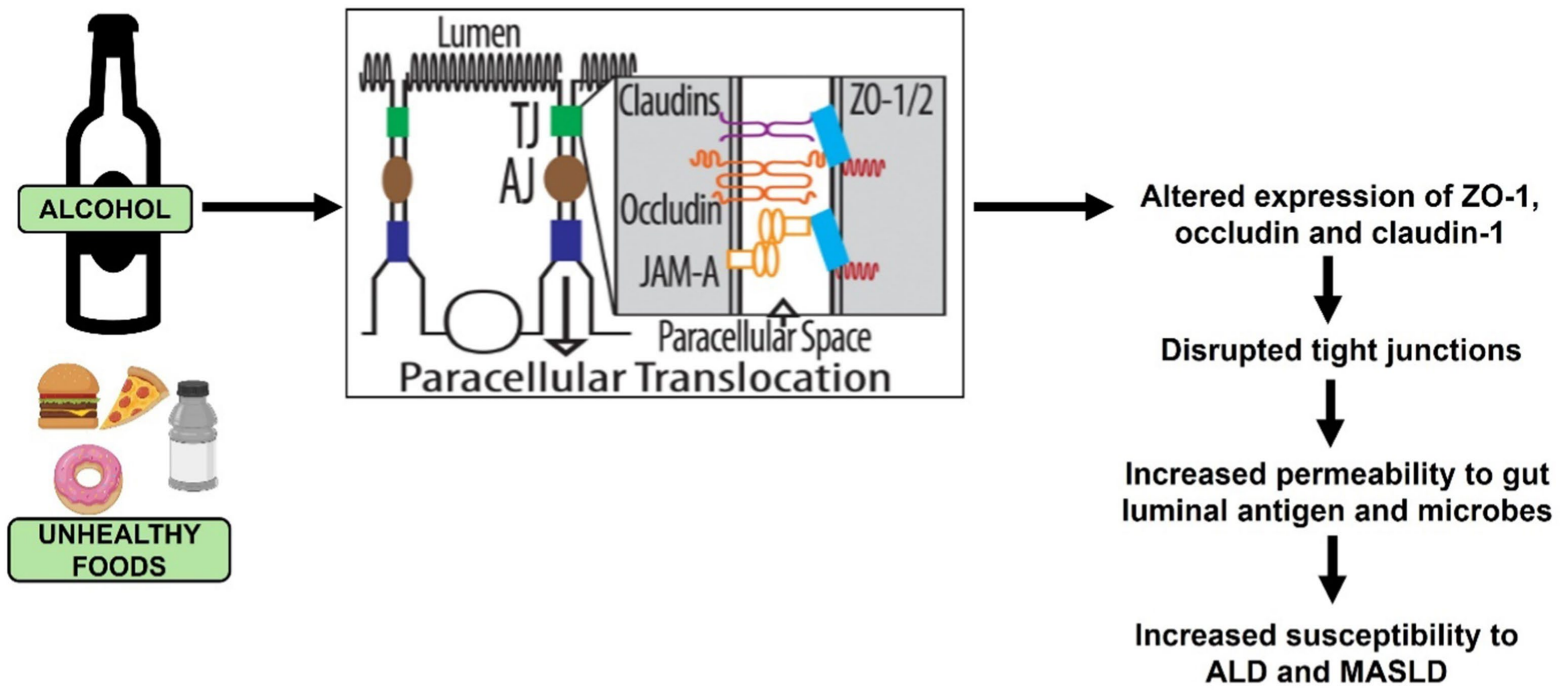
Ethanol and unhealthy food intake-induced tight junction (TJ) disruption that promotes the development of ALD and MASLD.

Several patient and animal studies have established that TJ disassembly occurs from altered expression of claudins, occludin and ZO1 (Figure 5), which promotes the pathogenesis of several diseases including ALD and MASLD (96–109).

In the context of ALD, ethanol and its primary metabolite, acetaldehyde, induce redistribution of TJ proteins to disrupt apical junctional complexes, facilitating increased intestinal permeability (34, 110–115). Studies have addressed the mechanisms by which ethanol and acetaldehyde disrupt these junctional complexes, but no unifying theory has yet been presented. It is clear, however, that alcohol/acetaldehyde alters post-translational modifications on the protein components of TJ that disrupt the formation and function of these apical junctional complexes (116–118). Another compelling evidence indicates that alcohol-induced intestinal TJ disruption is caused through elevations in intracellular levels of SAH, a detrimental metabolite of the methionine metabolic pathway (34). The importance of maintaining the barrier integrity is reinforced by reports that successful therapeutic interventions are associated with restoration of normal TJ protein expression (119). A recent study compared gut barrier dysfunction in patients with MASLD and ALD and reported more abnormalities in early stage of MASLD than in ALD (120).

**Figure 6:**
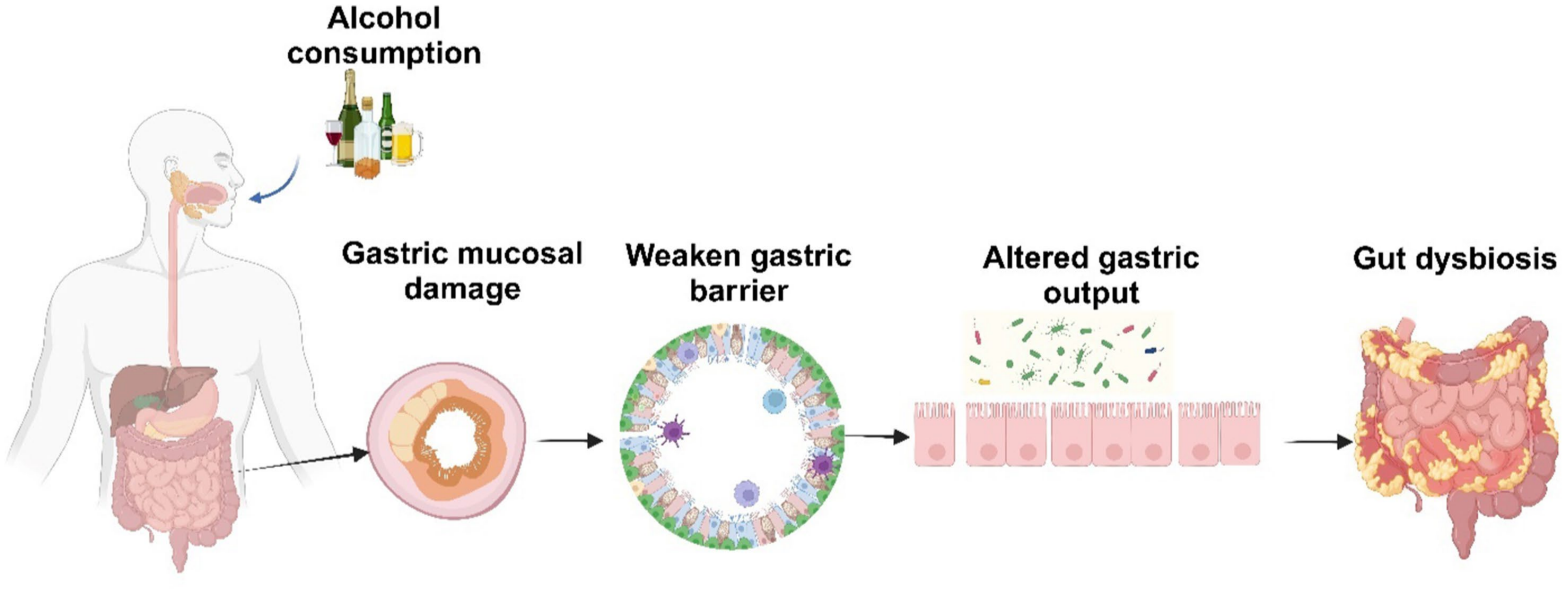
Alcohol-induced alterations in the gastric environment. Created with biorender.com.

Alcohol consumption stands as the primary cause of liver disease and influences the quantity and makeup of gut microbiota (121, 122). These imbalances or alterations in the gut microbiota composition and function are generally referred to as ‘dysbiosis’ (123). Alcohol intake, a Western diet high in animal fats and sugars, as well as factors such as bowel movement frequency, genetic predisposition, and disturbances in circadian rhythm, can increase the likelihood of dysbiosis (124). Ethanol acts as a modulator of the gastric microenvironment (Figure 6) through complex mechanisms, including altering gastric juice output, impairing gastric motility, damaging gut mucosa, weakening gastric barrier and disrupting mucosal defense (44, 125–127). Ethanol exposure also promotes the release of inflammatory and vasoactive substances, which can cause ischemia resulting in further mucosal damage (126). Alcohol by significantly changing the gastric microenvironment affects the composition of not only the gastric microbiota but also the microbes in the lower segments of the gastrointestinal tract. Interestingly, moderate alcohol use, particularly wine or beer, is linked to a lower risk of *Helicobacter pylori* infection (128). Conversely, alcohol consumption is associated with an increased risk of gastric cancer, especially in heavy drinkers (129). Alcohol misuse leads to bacterial overgrowth primarily in the upper small bowel, as reported in both preclinical models and human studies (130). This overgrowth results in qualitative changes in the intestinal microflora, dysbiosis in the cecum, and suppression of bactericidal proteins in the small intestine. The changes in alcohol-induced gut composition shows a decrease in beneficial bacteria and an increase in harmful bacteria. As summarized recently, alcohol increases the relative abundance of Proteobacteria, Fusobacteria, *Clostridium*, and *Lactococcus* and Enterobacteriaceae and decreases in that of Firmicutes and Bacteroidetes (131). Importantly, administrations of probiotics and their culture supernatants suppresses alcohol-induced increased intestinal permeability and endotoxemia in mice with ALD (132–134). In humans, the severity of ALD is linked to a specific microbiota signature, since fecal microbiota transplantation from patients with severe alcohol-associated hepatitis promotes liver inflammation in germ-free mice (135). Conversely, transplanting intestinal flora of a healthy donor to steroid-ineligible patients with hepatitis improved gut dysbiosis and clinical outcomes (136).

Furthermore, different drinking patterns, dose or experimental subjects may show varying effects and even completely different changes in the gut microbiota (93, 130, 137–144). Interestingly, alcohol consumption induces distinct changes in the gut composition that varies with short-term low-dose versus high-dose intake. In addition, while the acute alcohol-induced changes to the gut microbiota are found to be reversible (142), chronic alcohol consumption can lead to more drastic changes and have a more serious effect on the gut microbiota (137). The effects of short-term, low-dose alcohol consumption could be mitigated with appropriate probiotics and interventions like fermented rice liquors, red wine polyphenols or short chain fatty acid produced by protective gut bacteria (138, 141, 145, 146). Recovery from gut microbiota disruption caused by high-dose alcohol consumption requires longer and more complex interventions (146).

With regards to MASLD, the reader is referred to a recent review on changes in microbiota in MASLD patients (147). A noteworthy study revealed reduced gut microbiota diversity in MASLD patients compared to healthy individuals that leads to the disruption of the intestinal barrier in association with liver disease progression in the patient population (148). Furthermore, the severity of MASLD is linked to gut microbiota dysbiosis and alterations in the metabolic functions of the gastrointestinal microbiota. Notably, Bacteroidetes have been independently associated with MASH, while Ruminococcus is linked to significant fibrosis (149). Gut microbiota contribute to MASLD development through various mechanisms, including disruption of liver choline metabolism (necessary for VLDL synthesis and liver lipid export) (150), alterations in bile acid synthesis (151, 152), ethanol production (153), and increased lipopolysaccharides (LPS), which results in liver inflammation (154–156). These findings highlight the critical role of gut microbiota in the pathogenesis of MASLD and underscore the potential of microbiota-targeted therapies in managing the disease. Given the role of altered gut-liver axis in promoting liver ALD and MASLD, betaine has attracted attention as a potential therapeutic agent that supports gut health and protects the microbiome and intestinal barrier function (20, 157–159).

## Betaine, a functional nutrient

In recent years, there has been a global trend towards the use of natural substances existing in fruits, vegetables, and herbs as antioxidants and functional nutrients (20). One such compound is betaine (trimethylglycine), which is a short chain, non-essential amino acid derivative and a naturally occurring compound found in animals, plants, and microorganisms. Its rich dietary sources include seafood, especially marine invertebrates; wheat germ/bran; spinach and sugar beets (22, 160–162). While betaine can be obtained externally through nutrition (163), it is also endogenously generated through the metabolism of choline (164). Numerous organisms use betaine because of its vital biochemical roles, and they have developed distinct metabolic routes for both its biosynthesis and catabolism (20). This valuable compound has gained popularity as an ingredient in novel and functional foods due to its demonstrated health benefits. Cereals and cereal products are the main sources of betaine in human nutrition (165). Of the commonly consumed food, refined and whole grains are the best sources of betaine; however, there is 2–4 times higher betaine content in wholegrain products compared to refined grain products (165). Dietary betaine intake is generally less than 150 mg/day (165). Betaine can be synthesized chemically for use in secondary industries or extracted somewhat expensively from sugar beets or processed beet byproducts. Three types of commercial betaine are available: betaine hydrochloride, synthetic anhydrous betaine, and natural anhydrous betaine (157). Because natural betaine has better functional qualities than its synthetic counterpart, the pharmaceutical, cosmetic, and healthcare sectors prefer to employ it (157, 166).

Human plasma/serum typically contains 20–70 μmol/L betaine with the concentration being higher in the adult males than females (27). It is present in almost all tissues, with the highest concentrations in liver, kidney, and testes (28). Betaine insufficiency is associated with dysregulated lipid metabolism, diabetes, metabolic syndrome, and vascular diseases in patients (27, 28). Furthermore, patients with MASLD also exhibit betaine insufficiency (167). The depletion of hepatic betaine is also reported in animal models of ALD (168, 169). Betaine generally appears to be safe at a daily intake of 9–15 g (164, 170). Subacute and sub chronic rat studies determined that betaine is non-toxic at 1, 2 and 5% added to chow diet (171).

Betaine intake as a dietary supplement or through food has a similar bioavailability and is rapidly absorbed to participate in two main biological processes. In one, betaine acts as a donor of methyl groups in the methionine metabolic pathway (159, 172, 173) for remethylating and removing homocysteine (174, 175). In the second process, betaine acts as an osmolyte regulating cells’ adaptation to adverse osmotic environment and environmental stressors, such as low water levels, high salinity, and extreme temperatures (35, 176). In its role in remethylating homocysteine, betaine generates methionine, which upon enzymatic conversion to a key methyl donor, *S*-adenosylmethionine (SAM) is utilized in a variety of essential transmethylation reactions (22). The conversion of homocysteine to methionine is catalyzed by BHMT, an enzyme that was at first was thought to be present primarily in the liver (22). However, studies from several laboratory have reported the expression of BHMT in other important organs such as white adipose tissue and intestine (25, 34, 177). These results have prompted investigations into the potential health benefits of betaine (178). Several *BHMT* gene polymorphisms regulating its activity and protein level have been reported suggesting that loss-of-function polymorphisms by affecting betaine metabolism could adversely its efficacy in regulating homocysteine levels and related metabolic pathways (179, 180). However, studies on how genetic factors or population differences influence the efficiency of betaine utilization are few and limited to data collected from healthy population and only offer several speculative mechanisms of liver disease pathogenesis (179, 180). Interestingly, there is a complete loss of BHMT transcripts in liver tumors (181, 182).

### Betaine and liver

Research from our laboratory has demonstrated that betaine exerts its protective effects through its role in the BHMT-catalyzed reaction, which not only remethylates and removes homocysteine but also eliminates its detrimental precursor, SAH (21, 29, 30, 183–185). Thus, by preventing the ethanol-induced lowering of SAM: SAH ratio that impairs the activity of several crucial methyltransferase, betaine prevents the development of hallmark features of ALD such as steatosis, apoptosis, accumulation of altered proteins, lysosomal and proteasome dysfunction (24, 29, 30, 184–186). In relation to the gut-liver axis, betaine administration upregulates anti-oxidant defense system and prevents the ethanol-induced increases in serum endotoxin, alanine aminotransferase (AST), aspartate aminotransferase levels (ALT) and liver inflammatory factors, including TNF-*α*, IFN-γ, interleukin (IL)-18, cyclooxygenase-2 (COX-2), nitric oxide synthase 2 (NOS2), toll-like receptor 4 (TLR4), cluster of differentiation 14 (CD14) (187, 188). However, an earlier study using the intragastric ethanol-feeding model failed to show any protection by betaine feeding in the ethanol-induced up-regulation of CD14 and TNF-α expression (189). Using the same model, however, other laboratories have shown that betaine feeding prevented the alcohol-induced upregulation of several genes including TLR2/4, Janus kinase 3, α-2-macroglobulin (190) which implies that there are multiple pathways that betaine could act on to protect against the development of inflammation during ALD progression.

Similarly in the context of MASLD, studies have reported that betaine prevents the development of MASLD and reduces indices of liver damage in animal models and in patients (31, 32, 177, 191–195).

Readers are encouraged to refer to other comprehensive reviews on the protective effects of betaine on the liver in the context of ALD and MAFLD (20, 160, 192, 196, 197), as these aspects are not covered in detail here. Instead, this review focuses on the alterations in the gut-liver interaction, which has emerged as a crucial factor in the progression of advanced liver injury in both ALD and MASLD.

### Betaine and gut function

Intestinal enzyme activities are crucial for physiological processes (198). Betaine administration increases the activities of digestive enzymes, amylase, lipase, trypsin, and chymotrypsin, in the small intestine of stressed rats (35). Dietary betaine supplementation also increases the epithelial crypt-villus ratio in the guts of both healthy and challenged chicks, suggesting betaine’s protective role against coccidial infection is by stabilizing the mucosal structure (199). In addition to improving intestinal morphology and nutrient absorption in yellow-feathered broilers (200), dietary betaine reduces serum levels of the pro-inflammatory cytokines IL-1β, IL-6, TNF-*α*, and IFN-*γ* in goslings (201). Betaine not only regulates the osmotic microenvironment for microbes to survive but also provides extra sources of carbon and nitrogen for microbial growth. Furthermore, as a potent organic osmolyte, betaine can cooperate with endocrine hormones to regulate water and electrolyte balance and it plays an important role in improving intestinal functions by enhancing digestive enzymes, ameliorating damaged intestinal morphology, enriching intestinal microbiota (35) and enhancing intestinal barrier function (202).

### Betaine and intestinal barrier function

The direct protective effect of betaine on intestinal TJs and barrier integrity was revealed by an *in vitro* study using Caco-2 cells. The authors reported that co-treatment of these intestinal epithelial cells with betaine prevented both ethanol-and high SAH-induced TJ disruption to maintain epithelial barrier integrity (34). Many other studies have demonstrated the efficacy of betaine in other models of liver injury such as acute liver failure (ALF) showing that betaine protection occurred by an enhanced expression of TJ proteins, ZO-1 and occludin, thus modulating the TLR4 signaling pathway (203). Similarly, betaine treatment reversed LPS-induced downregulation of TJ proteins in intestinal porcine epithelial cells, thereby preventing barrier disruption (202). Furthermore, another study demonstrated that betaine pretreatment alleviates the inflammatory response and improves intestinal barrier function by enhancing the expression of TJ proteins, occludin and ZO-1 in a dextran sulfate sodium-induced colitis model (204). Overall, these studies highlight the significant therapeutic potential of betaine in protecting intestinal health by enhancing production of TJ proteins and their interaction, thereby mitigating gastrointestinal disorders and inflammatory diseases.

### Betaine and gut microbiota

Betaine administration changed the intestinal bacterial communities to promote the abundance of the commensals while reducing the pathogenic bacteria as shown in Table 1. There is yet no reports or publications related to modulation of the fungal or viral communities by betaine treatment in any animal models or patient studies.

**Table 1:** Protective role of betaine in regulating gut microbiome.

Physiological role	Affected microbial populations		References
	Increase	Decrease	
Betaine modulates the gut microbiota composition, promoting the growth of beneficial strains that produce short-chain fatty acids (SCFA)	*Prevotella, Ruminococcus, Oscillospira, Bifidobacterium, Akkermansia muciniphila, Lactobacillus*, and *Dorea*	*Desulfovibrio* and *Mucispirillum schaedleri*	(33)
Maternal betaine intake regulates gut microbiota and short chain fatty acids in offspring mice	*Desulfovibrio, Blautia*; *Romboutsia*	*Intestinimonas; Acetatifactor*	(233)
Betaine prevents Coccidiosis in avian species.		*Eimeria tenella*; *Eimeria acervulina*	(234)
Betaine strengthens microbial-associated phytoremediation process	Methylotrophic bacteria		(235)
Betaine improves intestinal functions by enhancing the digestive enzymes, ameliorating intestinal morphology, and enriching intestinal microbiota of high salt stressed rats.	*Romboutsia*; *Ruminiclostridium*		(35)
Betaine effectively improves intestinal injury in alcohol-associated liver disease animal model by inhibiting the TLR4/MyD88 signaling pathway, improving the intestinal mucosal barrier, and maintaining the gut microbiota composition	*Bacteroidaceae; Bacteroides; Parabacteroides; Prevotella*	*Coriobacteriaceae; Lachnospiraceae; Enterorhabdus; Coriobacteriales*	(203)
Betaine supplementation significantly improves growth performance, nutrient digestion, concentrations of total and individual volatile fatty acids, and total cecal beneficial bacterial count in rabbits	Total beneficial bacterial count	*Enterococcus* sp.; *E. coli*	(236)
Betaine supplementation improves gut health and supports the well-being of pullet chickens	*Lactobacillus agilis, Lactobacillus aviarius, Lactobacillus ingluviei, Lactobacillus johnsonii*, and *Lactobacillus saerimneri*		(237)

## Mechanism (s) of betaine protection against the development of chronic liver diseases

Several publications indicate that the mechanism of betaine protection against ALD and MASLD development is through remethylating homocysteine that by removing SAH preserves the methionine metabolic pathway and methylation pathways in the liver (20, 21, 24–26, 29, 30, 177, 183–185, 189, 191, 192, 195, 205–209). Despite extensive documentation on betaine’s effect on the liver, as shown in Table 2, only a few studies have been conducted that demonstrates its efficacy in preserving adipose and gut function in cell/animal models of MASLD or ALD. However, these limited studies also indicate that the protective effect of betaine is by preserving the methionine metabolic pathway via reducing SAH and/or homocysteine and rectifying the hypomethylation state (20, 34, 177, 210). Regarding gut-liver axis, several studies have indeed established that the trigger that causes systemic/liver inflammation to exacerbate liver injury (211) is prevented by betaine through stabilizing TJs between enterocytes and preventing the translocation of harmful gut-derived toxins/pathogens to the liver (34, 35, 202, 203). In addition, betaine modulates the gut microbiota, promoting a balanced microbial composition that supports healthy digestion and production of protective factors such as short chain fatty acids (SCFA), vitamins etc. (33, 212). These metabolites, in turn, regulate intestinal inflammation and barrier function (164, 213). Additional studies reveal that betaine influences bile acid metabolism, which is critical in regulating gut microbiota composition and intestinal permeability (214). This modulation helps maintain gut-liver homeostasis and reduces liver inflammation (203, 215). Another mechanism of betaine protection is by influencing gut osmolality and potentially altering intestinal pH, which enhancing digestive enzyme activity, restoring intestinal morphology, and increasing microbiota diversity (35). Further, this modulation of the microbiota can decrease the production of harmful metabolites and inflammatory mediators, thus preventing liver and systemic inflammation (13, 204).

**Table 2:** Protective role of betaine in various diseases/dysfunctional states.

Disease/Dysfunctional state	Model	Role	References
Ethanol-induced hepatic steatosis	Wister rats fed with Lieber-DeCarli control or ethanol diet	Betaine supplementation prevents and reverses alcohol-induced steatosis	(257)
		Betaine attenuates alcohol-associated steatosis by restoring phosphatidylcholine generation via the phosphatidylethanolamine methyltransferase pathway by normalizing hepatocellular *S*-adenosylmethionine (SAM) to S-adenosylhomocysteine (SAH) ratio	(29)
		Betaine prevents or blunts chronic ethanol-mediated alterations by normalizing SAM:SAH ratio to reduce hepatic triglycerides and CYP2E1 protein upregulation	(206, 208)
		Betaine administration corrects ethanol induced defective VLDL secretion, increased fat export from the liver and attenuates the development of alcohol-associated fatty liver	(185)
	Male Wistar rats’ drinks 5–25% water and ethanol	Betaine attenuates the alcohol-induced steatosis by improving hepatic lipid metabolism via upregulating PGC-1α and suppressing DGAT1, DGAT2, SREBP-1c, FAS, SREBP-2, and HMG-CoA reductase	(238)
	Guinea pigs fed with commercial chow diet	Betaine treatment decreases hepatic triglyceride, lipid peroxide levels and serum transaminase activities and increases GSH levels	(239)
Ethanol-induced hepatic apoptosis	Hepatocytes isolated from Wistar rats fed with Lieber-DeCarli control or ethanol diet,	Betaine prevents ethanol-induced rise in intracellular SAH levels, thereby mitigating alcohol-induced apoptosis by restoring normal methylation reactions	(26)
Ethanol-induced hepatic accumulation of damaged proteins	Wistar rats fed with Lieber-DeCarli control or ethanol diet	Betaine prevents ethanol-induced increase in accumulation of proteins bearing atypical isoaspartyl residues by normalizing SAM:SAH ratio to preserve the activity of the repair enzyme, protein-l-isoaspartyl methyltransferases	(30)
Ethanol-induced toxicity	Wistar rats fed with Lieber-DeCarli control or ethanol diet	Betaine prevents ethanol induced oxidative damage and peroxidative membrane injury in the brain as evident from a significant decrease in MDA, protein carbonyl levels and adenosine deaminase activities	(240)
Ethanol-induced hepatic oxidative stress	CYP2E1 over expressing HepG2 cells exposed to ethanol	Betaine reduces the oxidative stress induced HSP70 mRNA expression	(241)
Ethanol-induced adipose dysfunction and liver injury	Mice fed with Lieber-DeCarli control or ethanol diet	Betaine rectifies the impaired methylation status in adipose tissue, concomitant with attenuating lipolysis and alleviated alcohol-induced pathological changes in the liver	(210)
MASLD	Rats fed with high-fat diet	Betaine alleviates ROS-induced mitochondrial respiratory chain dysfunction	(242)
		Betaine protects by inhibiting high-mobility group box 1 and toll-like receptor 4 expression	(195)
	Mice fed with high-fat diet	Betaine reduces the high-fat diet induced fasting glucose and insulin, thereby improving insulin resistance, and preventing hepatic steatosis development	(191)
		Betaine enhances the conversion of white adipose tissue to brown adipose tissue through stimulated mitochondrial biogenesis	(243)
		Betaine supplementation alleviates hepatic pathological changes by attenuating insulin resistance and correcting abnormal adipokine (adiponectin, resistin, and leptin) production	(177)
		Maternal betaine intake regulates gut microbiota and short chain fatty acids and ameliorates hepatic steatosis in the offsprings	(233)
		Betaine modulates the gut microbiota composition by promoting the growth of beneficial strains that produce short chain fatty acids	(33)
		Maternal betaine supplementation inhibits hepatic NLRP3 inflammasome activation in the offspring	(244)
	Mice fed with high-sucrose diet	Betaine attenuates hepatic steatosis by increasing activation of hepatic AMP-activated protein kinase (AMPK) and attenuating lipogenic capability (enzyme activities and gene expression) in the liver	(193)
	Rats fed the methionine-and choline-deficient (MCD) diet	Betaine stimulates liver β-oxidation	(245)
	Mice fed the MCD diet	Betaine remethylates homocysteine, restores phosphatidylcholine generation and protect from oxidant stress	(246)
		Betaine reduces liver oxidant stress, inflammation, and apoptosis	(247)
	Clinical trials	Betaine attenuates steatosis, inflammation, and fibrosis	(192, 248–250)
MASH	Clinical trials	Betaine, improves hepatic function tests, homocysteine levels and histology in NASH patients	(192)
Methionine load	Human	Betaine lowers fasting plasma homocysteine levels and prevents a rise in plasma homocysteine levels after methionine intake	(251)
Homocystinuria	Humans	Betaine reduces plasma homocysteine concentrations and increases plasma methionine values	(252)
LPS-induced toxicity	White goslings	Betaine possesses anti-inflammation properties and improves intestinal barrier functions	(201)
	Intestinal porcine epithelial cells	Betaine attenuates LPS-induced downregulation of occludin and claudin-1 and restores the intestinal barrier function	(202)
	Caco-2 cells	Betaine pretreatment improves the inflammatory response and intestinal barrier function	(204)
	Cell cultures and animal models	Betaine independently reduces measures of oxidative damage, improves enterocyte health, as well as attenuates LPS-induced markers of liver damage and inflammatory responses	(163)
	Rats	Betaine intake attenuates the LPS-induced hepatotoxicity by preventing Kupffer cell activation and attenuating circulating TNF-α, ALT and AST levels	(253)
Inflammatory bowel disease	Dextran sodium sulfate-induced colitis in mice	Betaine attenuates colitis by regulating the inflammatory response, enhancing intestinal barrier function, and modulating gut microbiota composition	(204)
High salt stress	High salt stressed rats	Betaine improves intestinal function by enhancing the digestive enzymes, ameliorating intestinal morphology, and enriching intestinal microbiota. In addition, betaine, significantly improves markers of gut health (intestinal villi length and the ratio of villus height to crypt depth)	(35)
Infection	Coccidial infection in broiler chicks	Dietary betaine protects the jejunal villi against coccidial infection and stabilizes the mucosal structure in healthy broiler chicks	(199)
Heat stress	Meat-type ducks	Betaine supplementation increases body weight, and maintains blood pH and biochemical parameters (RBC count, hemoglobin, partial pressure of oxygen and carbon dioxide, Na^+^, K^+^, Cl^−^)	(254)
Minor hypertonic and thermal stress	Generic cell	Preserve cell volume, decrease long-term Na^+^ accumulation	(163)
Severe hypertonic and thermal stress	Generic cell	Decrease protein denaturation, decrease need for HSPs	
Restraint stress	Wister albino rats	Betaine inhibits the level of lipid peroxidation, corticosterone and protects the enzymatic antioxidant defense mechanisms	(170)
Stressful stimulus	Desert sheep and goats	Betaine ameliorates the endocrinological effects induced by the stressful stimulus	(255)
Anhedonia-like behavior (a key symptom of depression)	Mice subjected to chronic social defeat stress	Betaine modulates gut microbiota composition and associated metabolites	(256)

**Figure 7:**
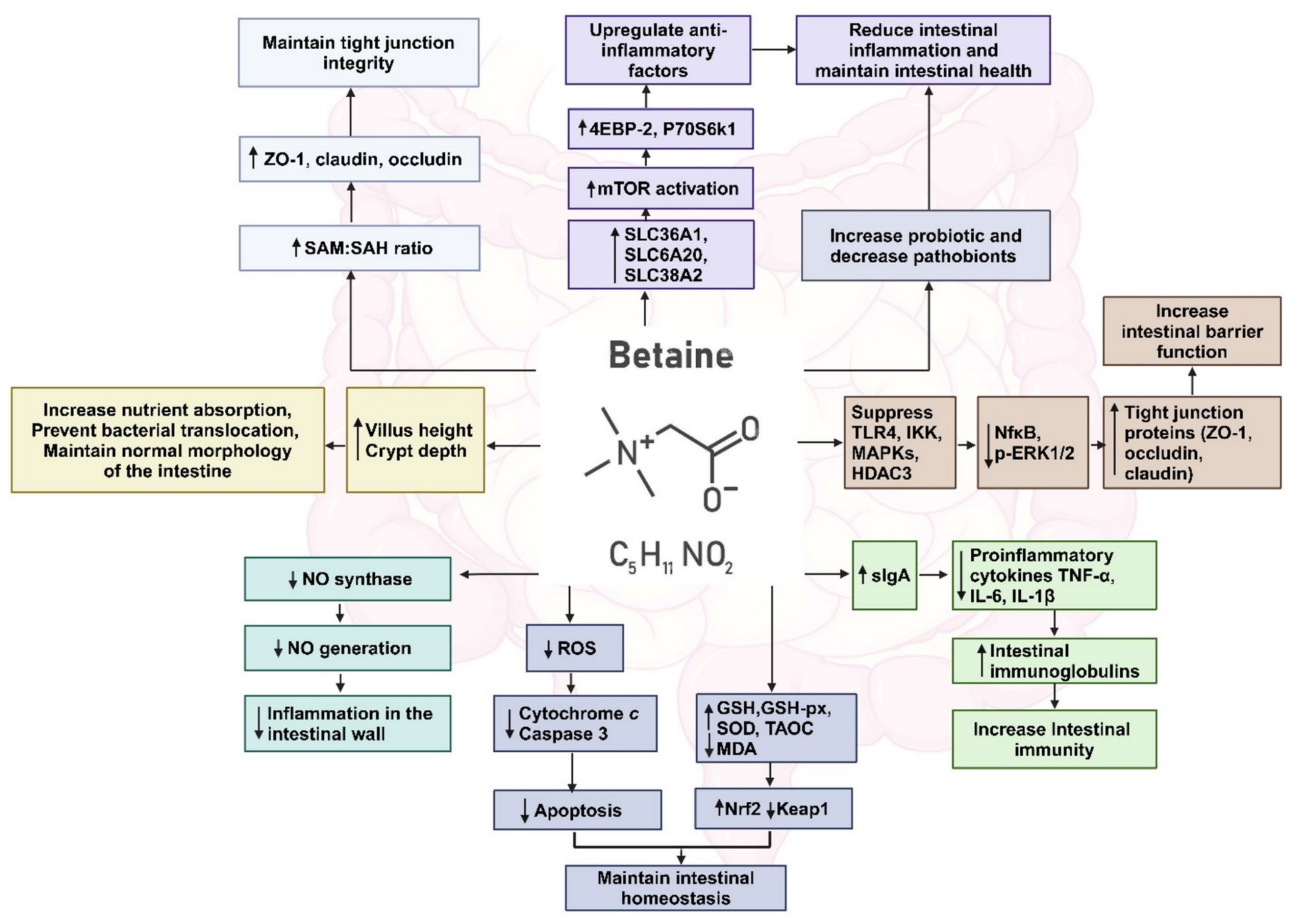
Multiple pathways by which betaine restores gut homeostasis. Created with biorender.com.

Betaine supplementation maintains the stability of the intestinal morphological structure by preventing cellular water loss (173), increasing the villus height, crypt depth and villus height/crypt depth ratio in the gut which prevent bacterial translocations (216). Betaine modulates the gut microbiota by increasing the abundance of commensals *Prevotella, Ruminococcus, Oscillospira, Bifidobacterium, Akkermansia muciniphila, Lactobacillus, Dorea, Bacteroidaceae, Bacteroides, Parabacteroides* and decrease the abundance of pathobionts including *Desulfovibrio, Mucispirillum schaedleri, Coriobacteriaceae, Lachnospiraceae, Enterorhabdus, Coriobacteriales* (33). Betaine increases the level of secretory immunoglobulin A (sIgA), which plays an important role in the clearance of pathogens and harmful substances, and also up-regulates IL-4 and down-regulated TNF-α in small intestinal mucosa (216). Betaine prevents nitric oxide (NO) generation markedly by inhibiting the expression of NO synthase, a principal enzyme for NO formation, consequently repressing inflammation in the intestinal wall (164). Betaine decreases malondialdehyde and increases glutathione and glutathione peroxidase activities in the small intestinal mucosa, thereby improving the antioxidant capacity of small intestine (217). Betaine supplementation up-regulates the gene expression of solute carrier family 7, member 6 (*SLC7A6*) solute carrier family 6, member 20 (*SLC6A20*), solute carrier family 38, member (*SLC38A2*) in the intestine. The consequent activation of mammalian target of rapamycin (mTOR), down-regulate the gene expression of pro-inflammatory factors by activating the phosphorylation state of downstream signaling factors 4EBP-2 and P70S6k1, to alleviate intestinal inflammation and maintain intestinal health (216). Figure 7 depicts the multiple pathways by which betaine modulates the gut homeostasis. While there is indication that the regulation of intestinal barrier function is through maintaining the methylation state (34), there has been no investigation till date on the mechanism by which betaine can maintain the commensal microbiota. Is it by regulating the intestinal pH, sIgA or by some other yet unknown mechanism? This area of research is highly underdeveloped. There is a need for characterizing how betaine regulates the gut microbiome and the further exploration of its role in maintaining intestinal barrier to prevent the development of not only common chronic liver diseases, but also inflammatory diseases of the gastrointestinal system.

## Betaine’s efficacy relative to other common therapeutic agents

Betaine exerts significant therapeutic and biological effects that are potentially beneficial for alleviating a diverse range of diseases (20). Probiotics have gained popularity in the last decade by its ability to restore the composition of the gut microbiome and introduce beneficial functions to gut microbial communities, resulting in the amelioration or prevention of gut inflammation and other intestinal or systemic disease phenotypes including liver diseases (218, 219). The major effects of probiotics include the restoration of commensal intestinal microbial communities, suppression of pathogenic bacterial communities, immunomodulation, stimulation of epithelial cell proliferation, and fortification of the intestinal barrier (220). These protective effects of probiotics enhance intestinal epithelial cell function and provide protection against physiological stress (220, 221). The most widely used probiotics like *Lactobacillus* and *Bifidobacterium* modulate gut microbiota composition and restore balance (222). Probiotics also enhance SCFA production and improve gut barrier integrity, thereby reducing systemic inflammation (33). Hajirezaee et al. (223) reported significant growth enhancement, digestive enzyme activities, antioxidant capacity, and immune performance in fish receiving a combination of the probiotic *Lactobacillus rhamnosus* and betaine therapy. In comparison with probiotics, betaine alone can directly protect liver, adipose, intestinal barrier integrity and restore the commensal microbiota and SCFA production (33) and thus in comparison functions to prevent liver disease and other inflammatory condition without any adjunct probiotic use.

Antioxidants like *N*-acetylcysteine and glutathione effectively mitigate oxidative stress and inflammation, primarily through scavenging reactive oxygen species (224, 225). However, betaine also possesses antioxidant properties by not only reducing oxidative stress via homocysteine remethylation but also by stabilizing the gut barrier and reducing LPS-mediated inflammation (164). Unlike probiotics or traditional antioxidants, betaine addresses both gut microbiota modulation and systemic inflammation through dual pathways: enhancing methylation processes and regulating osmoregulation in gut epithelial cells. This dual role is especially critical in the gut-liver axis, where betaine prevents endotoxin translocation and reduces hepatic inflammation (20).

There are many studies that have illustrated the therapeutic potential of betaine in combination with other compounds for improving health outcomes in both animals and human studies across a range of conditions ranging from inflammation, metabolic stressors, infections to metabolic regulation and immune support (226–232). While these combination therapies exploited betaine’s efficacy in reducing oxidative stress, preventing inflammation and, enhancing methylation processes to improve outcomes, none were related to maintaining gut-liver interaction to prevent chronic liver diseases. Hence discussion of these combinational therapies is beyond the scope of this review.

## Limitations and challenges of using betaine as a therapeutic agent

Betaine helps to maintain health levels of stomach acids, increase nutrient absorption and support digestion. Betaine helps to manage harmful microbes and reduce the bacterial infections in the gut. Betaine reduces insulin resistance, lipid synthesis, inflammation, endoplasmic reticulum stress, hepatic oxidative stress, promoting fatty acid oxidation, reshape the intestinal microbiota, prevent liver steatosis. Despite the efficacy of betaine in multiple preclinical studies in promoting overall health by its effect in the gut, liver, adipose, there are several limitations and challenges. One minor challenge is that betaine supplementation causes gastrointestinal discomfort, including bloating or diarrhea in some individuals, particularly when consumed in high amounts. In addition to these mild adverse events, it is likely that loss-of-function *BHMT* polymorphisms may also pose a challenge in conducting human trials. However, prior selection of the participants can overcome this obstacle. The biggest challenge in its use in clinical settings is the lack of interest by pharmaceuticals to fund these studies. This is because betaine is easily accessible, orally bioavailable, very soluble and a naturally occurring metabolite in our body. It therefore falls on the investigator-initiated efforts to solicit funds for conducting robust clinical trials to validate betaine efficacy outcomes in individuals with inherent differences in gut microbiota composition, dietary habits, metabolic profiles, and liver disease severity. Further, collaborative research involving multi-omics approaches (genomics, metabolomics, and microbiomics) could provide insights into individual variability and enhance the predictive power of preclinical findings. Future research must address these limitations through well-designed studies exploring betaine dose, molecular mechanisms, and long-term safety to harness its potential in promoting gut and overall health in preventing chronic liver diseases.

Despite the challenges mentioned above, there are a few randomized clinical trials conducted using betaine supplementation demonstrating its potential therapeutic applications in a range of diseases and conditions such as cardiovascular diseases, metabolic disorder during pregnancy, inflammatory disorders etc. (NCT01371357, NCT00102843, NCT00126347, NCT01950039, NCT04633044, and NCT06042270). However, none of these clinical trials were related to gut-liver axis modulation in relation to chronic liver diseases, hence no details are included in this review. The reader can refer to the NCT numbers for details on these clinical trials and outcomes.

## Translation from preclinical findings to clinical applications

Despite the limitations and challenges, the development of therapeutic protocols to ensure the translational potential of betaine from preclinical to clinical studies are important. The primary goal is to conduct advanced preclinical studies using diverse animal model studies that mimic human conditions to understand the mechanism of betaine’s protective effect on not only gut biology but also other organ systems such as the brain. Another important goal is to validate the preclinical data by (1) conducting well organized early phase clinical trials to assess safety, optimal dosages, and initial efficacy of betaine in humans; (2) incorporating different layers of patient criteria such as genetic polymorphisms, gut microbiota composition and metabolic profiles to understand how individual differences may affect betaine’s efficacy; and (3) conducting long-term observational studies and randomized controlled trials in diverse populations to validate betaine’s effectiveness in modulating gut-liver axis for treating chronic liver and other inflammation-related diseases. These steps will bridge the gap between preclinical research and clinical application of betaine in preventing the development of chronic liver diseases.

## Conclusion

In conclusion, gastrointestinal alterations play a significant role in the development and progression of chronic liver diseases such as ALD and MASLD. Disruption of gut homeostasis, including alterations in gut microbiota composition, impaired intestinal barrier function and increased microbial translocation, initiates a cascade of immune activation and inflammatory responses that promotes advanced liver damage. Many therapeutic approaches targeting chronic liver diseases focus on modulating gut dysbiosis and enhancing barrier function. Betaine, through its ability to regulate the protective gut microbiota and maintain intestinal barrier integrity, emerges as a promising therapeutic agent for preventing the development of chronic liver diseases, including ALD and MASLD. Betaine protection is mediated via donating a methyl group in methionine metabolic pathway to remove homocysteine and SAH and preserves cellular methylation potential, thereby safeguarding the gut microbiota and intestinal barrier function. Further exploration of the molecular mechanisms of betaine action in preclinical and clinical studies will enhance our understanding of its therapeutic potential and pave the way for targeted interventions in the management of liver diseases and other inflammatory bowel diseases or systemic inflammatory conditions.

## References

[1] Albuquerque-Souza E Sahingur SE. Periodontitis, chronic liver diseases, and the emerging oral-gut-liver axis. Periodontol. (2022) 89:125–41. doi: 10.1111/prd.12427, PMID: 35244954 PMC9314012

[2] Asrani SK Devarbhavi H Eaton J Kamath PS. Burden of liver diseases in the world. J Hepatol. (2019) 70:151–71. doi: 10.1016/j.jhep.2018.09.014 30266282

[3] Trefts E Gannon M Wasserman DH. The liver. Curr Biol. (2017) 27:R1147–51. doi: 10.1016/j.cub.2017.09.019, PMID: 29112863 PMC5897118

[4] Alvarez-Mercado AI Navarro-Oliveros M Robles-Sanchez C Plaza-Diaz J Saez-Lara MJ Munoz-Quezada S. Microbial population changes and their relationship with human health and disease. Microorganisms. (2019) 7:68. doi: 10.3390/microorganisms7030068, PMID: 30832423 PMC6463060

[5] Cornide-Petronio ME Alvarez-Mercado AI Jimenez-Castro MB Peralta C. Current knowledge about the effect of nutritional status, supplemented nutrition diet, and gut microbiota on hepatic ischemia-reperfusion and regeneration in liver surgery. Nutrients. (2020) 12:284. doi: 10.3390/nu12020284 31973190 PMC7071361

[6] Petronini PG De Angelis EM Borghetti P Borghetti AF Wheeler KP. Modulation by betaine of cellular responses to osmotic stress. Biochem J. (1992) 282:69–73. doi: 10.1042/bj2820069, PMID: 1311562 PMC1130890

[7] Sharma A Nagalli S. Chronic liver disease In: Stat pearls. Treasure Island, FL: StatPearls Publishing LLC. (2024). 32119484

[8] Trovato GM Catalano D Martines GF Pirri C Trovato FM. Western dietary pattern and sedentary life: independent effects of diet and physical exercise intensity on NAFLD. Am J Gastroenterol. (2013) 108:1932–3. doi: 10.1038/ajg.2013.356, PMID: 24300872

[9] Albillos A De Gottardi A Rescigno M. The gut-liver axis in liver disease: pathophysiological basis for therapy. J Hepatol. (2020) 72:558–77. doi: 10.1016/j.jhep.2019.10.003, PMID: 31622696

[10] Konturek PC Harsch IA Konturek K Schink M Konturek T Neurath MF. Gut(−)liver Axis: how Do gut Bacteria influence the liver? Med Sci. (2018) 6:79. doi: 10.3390/medsci6030079 PMC6165386 30227645

[11] Song Q Zhang X. The role of gut-liver axis in gut microbiome dysbiosis associated NAFLD and NAFLD-HCC. Biomedicines. (2022) 10:524. doi: 10.3390/biomedicines10030524, PMID: 35327326 PMC8945287

[12] Stolfi C Maresca C Monteleone G Laudisi F. Implication of intestinal barrier dysfunction in gut Dysbiosis and diseases. Biomedicines. (2022) 10:289. doi: 10.3390/biomedicines10020289, PMID: 35203499 PMC8869546

[13] Wang L Cao ZM Zhang LL Li JM Lv WL. The role of gut microbiota in some liver diseases: from an immunological perspective. Front Immunol. (2022) 13:923599. doi: 10.3389/fimmu.2022.923599, PMID: 35911738 PMC9326173

[14] Nicoletti A Ponziani FR Biolato M Valenza V Marrone G Sganga G. Intestinal permeability in the pathogenesis of liver damage: from non-alcoholic fatty liver disease to liver transplantation. World J Gastroenterol. (2019) 25:4814–34. doi: 10.3748/wjg.v25.i33.4814, PMID: 31543676 PMC6737313

[15] Artis D. Epithelial-cell recognition of commensal bacteria and maintenance of immune homeostasis in the gut. Nat Rev Immunol. (2008) 8:411–20. doi: 10.1038/nri2316, PMID: 18469830

[16] Varol C Zigmond E Jung S. Securing the immune tightrope: mononuclear phagocytes in the intestinal lamina propria. Nat Rev Immunol. (2010) 10:415–26. doi: 10.1038/nri2778, PMID: 20498668

[17] Crispe IN. The liver as a lymphoid organ. Annu Rev Immunol. (2009) 27:147–63. doi: 10.1146/annurev.immunol.021908.132629, PMID: 19302037

[18] Wiest R Albillos A Trauner M Bajaj JS Jalan R. Targeting the gut-liver axis in liver disease. J Hepatol. (2017) 67:1084–103. doi: 10.1016/j.jhep.2017.05.007 28526488

[19] Yang X Lu D Zhuo J Lin Z Yang M Xu X. The gut-liver Axis in immune remodeling: new insight into liver diseases. Int J Biol Sci. (2020) 16:2357–66. doi: 10.7150/ijbs.46405, PMID: 32760203 PMC7378637

[20] Arumugam MK Paal MC Donohue TM Ganesan M Osna NA Kharbanda KK. Beneficial effects of betaine: a comprehensive review. Biology. (2021) 10:456. doi: 10.3390/biology10060456, PMID: 34067313 PMC8224793

[21] Barak AJ Beckenhauer HC Mailliard ME Kharbanda KK Tuma DJ. Betaine lowers elevated s-adenosylhomocysteine levels in hepatocytes from ethanol-fed rats. J Nutr. (2003) 133:2845–8. doi: 10.1093/jn/133.9.2845, PMID: 12949375

[22] Craig SA. Betaine in human nutrition. Am J Clin Nutr. (2004) 80:539–49. doi: 10.1093/ajcn/80.3.539, PMID: 15321791

[23] Ji C Shinohara M Kuhlenkamp J Chan C Kaplowitz N. Mechanisms of protection by the betaine-homocysteine methyltransferase/betaine system in HepG2 cells and primary mouse hepatocytes. Hepatology. (2007) 46:1586–96. doi: 10.1002/hep.21854, PMID: 17705221 PMC2642650

[24] Arumugam MK Chava S Perumal SK Paal MC Rasineni K Ganesan M. Acute ethanol-induced liver injury is prevented by betaine administration. Front Physiol. (2022) 13:940148. doi: 10.3389/fphys.2022.940148, PMID: 36267591 PMC9577233

[25] Arumugam MK Chava S Rasineni K Paal MC Donohue TM Jr Osna NA. Elevated S-adenosylhomocysteine induces adipocyte dysfunction to promote alcohol-associated liver steatosis. Sci Rep. (2021) 11:14693. doi: 10.1038/s41598-021-94180-x, PMID: 34282217 PMC8289835

[26] Kharbanda KK Rogers DD 2nd Mailliard ME Siford GL Barak AJ Beckenhauer HC. Role of elevated S-adenosylhomocysteine in rat hepatocyte apoptosis: protection by betaine. Biochem Pharmacol. (2005) 70:1883–90. doi: 10.1016/j.bcp.2005.09.021 16253211

[27] Lever M Slow S. The clinical significance of betaine, an osmolyte with a key role in methyl group metabolism. Clin Biochem. (2010) 43:732–44. doi: 10.1016/j.clinbiochem.2010.03.009, PMID: 20346934

[28] Slow S Lever M Chambers ST George PM. Plasma dependent and independent accumulation of betaine in male and female rat tissues. Physiol Res. (2009) 58:403–10. doi: 10.33549/physiolres.931569 18637704

[29] Kharbanda KK Mailliard ME Baldwin CR Beckenhauer HC Sorrell MF Tuma DJ. Betaine attenuates alcoholic steatosis by restoring phosphatidylcholine generation via the phosphatidylethanolamine methyltransferase pathway. J Hepatol. (2007) 46:314–21. doi: 10.1016/j.jhep.2006.08.024, PMID: 17156888

[30] Kharbanda KK Mailliard ME Baldwin CR Sorrell MF Tuma DJ. Accumulation of proteins bearing atypical isoaspartyl residues in livers of alcohol-fed rats is prevented by betaine administration: effects on protein-L-isoaspartyl methyltransferase activity. J Hepatol. (2007) 46:1119–25. doi: 10.1016/j.jhep.2007.01.026, PMID: 17336420

[31] Patrick L. Non-alcoholic fatty liver disease: relationship to insulin sensitivity and oxidative stress. Treatment approaches using vitamin E, magnesium, and betaine. Altern Med Rev. (2002) 7:276–91. 12197781

[32] Xu L Huang D Hu Q Wu J Wang Y Feng J. Betaine alleviates hepatic lipid accumulation via enhancing hepatic lipid export and fatty acid oxidation in rats fed with a high-fat diet. Br J Nutr. (2015) 113:1835–43. doi: 10.1017/S0007114515001130, PMID: 25920593

[33] Du J Zhang P Luo J Shen L Zhang S Gu H. Dietary betaine prevents obesity through gut microbiota-drived microRNA-378a family. Gut Microbes. (2021) 13:1–19. doi: 10.1080/19490976.2020.1862612, PMID: 33550882 PMC7889173

[34] Thomes PG Osna NA Bligh SM Tuma DJ Kharbanda KK. Role of defective methylation reactions in ethanol-induced dysregulation of intestinal barrier integrity. Biochem Pharmacol. (2015) 96:30–8. doi: 10.1016/j.bcp.2015.04.018, PMID: 25931143

[35] Wang H Li S Fang S Yang X Feng J. Betaine improves intestinal functions by enhancing digestive enzymes, ameliorating intestinal morphology, and enriching intestinal microbiota in high-salt stressed rats. Nutrients. (2018) 10:907. doi: 10.3390/nu10070907 30012963 PMC6073560

[36] Chen M Zhong W Xu W. Alcohol and the mechanisms of liver disease. J Gastroenterol Hepatol. (2023) 38:1233–40. doi: 10.1111/jgh.16282, PMID: 37423758

[37] Torruellas C French SW Medici V. Diagnosis of alcoholic liver disease. World J Gastroenterol. (2014) 20:11684–99. doi: 10.3748/wjg.v20.i33.11684, PMID: 25206273 PMC4155359

[38] Wu X Fan X Miyata T Kim A Cajigas-Du Ross CK Ray S. Recent advances in understanding of pathogenesis of alcohol-associated liver disease. Annu Rev Pathol. (2023) 18:411–38. doi: 10.1146/annurev-pathmechdis-031521-030435, PMID: 36270295 PMC10060166

[39] Elamin E Masclee A Juuti-Uusitalo K Van Ijzendoorn S Troost F Pieters HJ. Fatty acid ethyl esters induce intestinal epithelial barrier dysfunction via a reactive oxygen species-dependent mechanism in a three-dimensional cell culture model. PLoS One. (2013) 8:e58561. doi: 10.1371/journal.pone.0058561, PMID: 23526996 PMC3602318

[40] Elamin EE Masclee AA Dekker J Jonkers DM. Ethanol metabolism and its effects on the intestinal epithelial barrier. Nutr Rev. (2013) 71:483–99. doi: 10.1111/nure.12027, PMID: 23815146

[41] Mackowiak B Fu Y Maccioni L Gao B. Alcohol-associated liver disease. J Clin Invest. (2024) 134:6345. doi: 10.1172/JCI176345, PMID: 38299591 PMC10836812

[42] Seth D Haber PS Syn WK Diehl AM Day CP. Pathogenesis of alcohol-induced liver disease: classical concepts and recent advances. J Gastroenterol Hepatol. (2011) 26:1089–105. doi: 10.1111/j.1440-1746.2011.06756.x, PMID: 21545524

[43] Lieber CS. Role of oxidative stress and antioxidant therapy in alcoholic and non-alcoholic liver diseases. Adv Pharmacol. (1997) 38:601–28. PMID: 8895826 10.1016/s1054-3589(08)61001-7

[44] Rocco A Compare D Angrisani D Sanduzzi Zamparelli M Nardone G. Alcoholic disease: liver and beyond. World J Gastroenterol. (2014) 20:14652–9. doi: 10.3748/wjg.v20.i40.14652, PMID: 25356028 PMC4209531

[45] Osna NA Donohue TM Jr Kharbanda KK. Alcoholic liver disease: pathogenesis and current management. Alcohol Res. (2017) 38:147–61. PMID: 28988570 10.35946/arcr.v38.2.01 PMC5513682

[46] Casanova J Bataller R. Alcoholic hepatitis: prognosis and treatment. Gastroenterol Hepatol. (2014) 37:262–8. doi: 10.1016/j.gastrohep.2014.02.001, PMID: 24656653

[47] Chacko KR Reinus J. Spectrum of alcoholic liver disease. Clin Liver Dis. (2016) 20:419–27. doi: 10.1016/j.cld.2016.02.002, PMID: 27373606

[48] Prado V Caballeria J Vargas V Bataller R Altamirano J. Alcoholic hepatitis: how far are we and where are we going? Ann Hepatol. (2016) 15:463–73. doi: 10.5604/16652681.1202885 PMID: 27236145

[49] Gopal T Ai W Casey CA Donohue TM Jr Saraswathi V. A review of the role of ethanol-induced adipose tissue dysfunction in alcohol-associated liver disease. Alcohol Clin Exp Res. (2021) 45:1927–39. doi: 10.1111/acer.14698, PMID: 34558087 PMC9153937

[50] Hsu CL Schnabl B. The gut-liver axis and gut microbiota in health and liver disease. Nat Rev Microbiol. (2023) 21:719–33. doi: 10.1038/s41579-023-00904-3, PMID: 37316582 PMC10794111

[51] Luo L Chang Y Sheng L. Gut-liver axis in the progression of non-alcoholic fatty liver disease: from the microbial derivatives-centered perspective. Life Sci. (2023) 321:121614. doi: 10.1016/j.lfs.2023.121614, PMID: 36965522

[52] Maccioni L Gao B Leclercq S Pirlot B Horsmans Y De Timary P. Intestinal permeability, microbial translocation, changes in duodenal and fecal microbiota, and their associations with alcoholic liver disease progression in humans. Gut Microbes. (2020) 12:1782157. doi: 10.1080/19490976.2020.1782157, PMID: 32588725 PMC7524402

[53] Mak KM Kee D Shin DW. Alcohol-associated capillarization of sinusoids: a critique since the discovery by Schaffner and popper in 1963. Anat Rec (Hoboken). (2022) 305:1592–610. doi: 10.1002/ar.24829 34766732

[54] Bessone F Razori MV Roma MG. Molecular pathways of non-alcoholic fatty liver disease development and progression. Cell Mol Life Sci. (2019) 76:99–128. doi: 10.1007/s00018-018-2947-0, PMID: 30343320 PMC11105781

[55] Angulo P. Non-alcoholic fatty liver disease. N Engl J Med. (2002) 346:1221–31. doi: 10.1056/NEJMra011775, PMID: 11961152

[56] Younossi Z Anstee QM Marietti M Hardy T Henry L Eslam M. Global burden of NAFLD and NASH: trends, predictions, risk factors and prevention. Nat Rev Gastroenterol Hepatol. (2018) 15:11–20. doi: 10.1038/nrgastro.2017.109, PMID: 28930295

[57] Younossi ZM Koenig AB Abdelatif D Fazel Y Henry L Wymer M. Global epidemiology of non-alcoholic fatty liver disease-Meta-analytic assessment of prevalence, incidence, and outcomes. Hepatology. (2016) 64:73–84. doi: 10.1002/hep.28431, PMID: 26707365

[58] Musso G Gambino R De Michieli F Cassader M Rizzetto M Durazzo M. Dietary habits and their relations to insulin resistance and postprandial lipemia in non-alcoholic steatohepatitis. Hepatology. (2003) 37:909–16. doi: 10.1053/jhep.2003.50132, PMID: 12668986

[59] Clark JM Brancati FL Diehl AM. The prevalence and etiology of elevated aminotransferase levels in the United States. Am J Gastroenterol. (2003) 98:960–7. doi: 10.1111/j.1572-0241.2003.07486.x 12809815

[60] Guo X Yin X Liu Z Wang J. Non-alcoholic fatty liver disease (NAFLD) pathogenesis and natural products for prevention and treatment. Int J Mol Sci. (2022) 23:5489. doi: 10.3390/ijms232415489, PMID: 36555127 PMC9779435

[61] Singh S Osna NA Kharbanda KK. Treatment options for alcoholic and non-alcoholic fatty liver disease: a review. World J Gastroenterol. (2017) 23:6549–70. doi: 10.3748/wjg.v23.i36.6549, PMID: 29085205 PMC5643281

[62] Leoni S Tovoli F Napoli L Serio I Ferri S Bolondi L. Current guidelines for the management of non-alcoholic fatty liver disease: a systematic review with comparative analysis. World J Gastroenterol. (2018) 24:3361–73. doi: 10.3748/wjg.v24.i30.3361, PMID: 30122876 PMC6092580

[63] Sheka AC Adeyi O Thompson J Hameed B Crawford PA Ikramuddin S. Non-alcoholic steatohepatitis: a review. JAMA. (2020) 323:1175–83. doi: 10.1001/jama.2020.2298 32207804

[64] Day CP James OF. Steatohepatitis: a tale of two “hits?”. Gastroenterology. (1998) 114:842–5. doi: 10.1016/S0016-5085(98)70599-2, PMID: 9547102

[65] Aubert J Begriche K Knockaert L Robin MA Fromenty B. Increased expression of cytochrome P450 2E1 in non-alcoholic fatty liver disease: mechanisms and pathophysiological role. Clin Res Hepatol Gastroenterol. (2011) 35:630–7. doi: 10.1016/j.clinre.2011.04.015, PMID: 21664213

[66] Arslan N. Obesity, fatty liver disease and intestinal microbiota. World J Gastroenterol. (2014) 20:16452–63. doi: 10.3748/wjg.v20.i44.16452, PMID: 25469013 PMC4248188

[67] Brunt EM. Grading and staging the histopathological lesions of chronic hepatitis: the Knodell histology activity index and beyond. Hepatology. (2000) 31:241–6. doi: 10.1002/hep.510310136, PMID: 10613753

[68] Wree A Broderick L Canbay A Hoffman HM Feldstein AE. From NAFLD to NASH to cirrhosis-new insights into disease mechanisms. Nat Rev Gastroenterol Hepatol. (2013) 10:627–36. doi: 10.1038/nrgastro.2013.149, PMID: 23958599

[69] Pabst O Hornef MW Schaap FG Cerovic V Clavel T Bruns T. Gut-liver axis: barriers and functional circuits. Nat Rev Gastroenterol Hepatol. (2023) 20:447–61. doi: 10.1038/s41575-023-00771-6, PMID: 37085614

[70] Ramadori G Moriconi F Malik I Dudas J. Physiology and pathophysiology of liver inflammation, damage and repair. J Physiol Pharmacol. (2008) 59:107–17. 18802219

[71] Tilg H Adolph TE Trauner M. Gut-liver axis: pathophysiological concepts and clinical implications. Cell Metab. (2022) 34:1700–18. doi: 10.1016/j.cmet.2022.09.017, PMID: 36208625

[72] Seki E Schnabl B. Role of innate immunity and the microbiota in liver fibrosis: crosstalk between the liver and gut. J Physiol. (2012) 590:447–58. doi: 10.1113/jphysiol.2011.219691, PMID: 22124143 PMC3379693

[73] Tsuchida T Friedman SL. Mechanisms of hepatic stellate cell activation. Nat Rev Gastroenterol Hepatol. (2017) 14:397–411. doi: 10.1038/nrgastro.2017.38, PMID: 28487545

[74] Verbeke L Nevens F Laleman W. Bench-to-beside review: acute-on-chronic liver failure – linking the gut, liver and systemic circulation. Crit Care. (2011) 15:233. doi: 10.1186/cc10424, PMID: 22104633 PMC3334742

[75] Rao R. Endotoxemia and gut barrier dysfunction in alcoholic liver disease. Hepatology. (2009) 50:638–44. doi: 10.1002/hep.23009, PMID: 19575462 PMC6209509

[76] Raya Tonetti F Eguileor A Mrdjen M Pathak V Travers J Nagy LE. Gut-liver axis: recent concepts in pathophysiology in alcohol-associated liver disease. Hepatology. (2024) 80:1342–71. doi: 10.1097/HEP.0000000000000924, PMID: 38691396 PMC11801230

[77] Shao T Zhao C Li F Gu Z Liu L Zhang L. Intestinal HIF-1alpha deletion exacerbates alcoholic liver disease by inducing intestinal dysbiosis and barrier dysfunction. J Hepatol. (2018) 69:886–95. doi: 10.1016/j.jhep.2018.05.021, PMID: 29803899 PMC6615474

[78] Starkel P Leclercq S De Timary P Schnabl B. Intestinal dysbiosis and permeability: the yin and yang in alcohol dependence and alcoholic liver disease. Clin Sci. (2018) 132:199–212. doi: 10.1042/CS20171055, PMID: 29352076

[79] Luissint AC Parkos CA Nusrat A. Inflammation and the intestinal barrier: leukocyte-epithelial cell interactions, cell junction remodeling, and mucosal repair. Gastroenterology. (2016) 151:616–32. doi: 10.1053/j.gastro.2016.07.008, PMID: 27436072 PMC5317033

[80] Van Itallie CM Anderson JM. Architecture of tight junctions and principles of molecular composition. Semin Cell Dev Biol. (2014) 36:157–65. doi: 10.1016/j.semcdb.2014.08.011, PMID: 25171873 PMC4254347

[81] Hugon P Dufour JC Colson P Fournier PE Sallah K Raoult D. A comprehensive repertoire of prokaryotic species identified in human beings. Lancet Infect Dis. (2015) 15:1211–9. doi: 10.1016/S1473-3099(15)00293-5, PMID: 26311042

[82] Pastorelli L De Salvo C Mercado JR Vecchi M Pizarro TT. Central role of the gut epithelial barrier in the pathogenesis of chronic intestinal inflammation: lessons learned from animal models and human genetics. Front Immunol. (2013) 4:280. doi: 10.3389/fimmu.2013.00280, PMID: 24062746 PMC3775315

[83] Assimakopoulos SF Triantos C Thomopoulos K Fligou F Maroulis I Marangos M. Gut-origin sepsis in the critically ill patient: pathophysiology and treatment. Infection. (2018) 46:751–60. doi: 10.1007/s15010-018-1178-5, PMID: 30003491

[84] Anderson JM Van Itallie CM. Tight junctions and the molecular basis for regulation of paracellular permeability. Am J Phys. (1995) 269:G467–75. doi: 10.1152/ajpgi.1995.269.4.G467, PMID: 7485497

[85] Heinemann U Schuetz A. Structural features of tight-junction proteins. Int J Mol Sci. (2019) 20:6020. doi: 10.3390/ijms20236020, PMID: 31795346 PMC6928914

[86] Turner JR. Intestinal mucosal barrier function in health and disease. Nat Rev Immunol. (2009) 9:799–809. doi: 10.1038/nri2653, PMID: 19855405

[87] Zihni C Mills C Matter K Balda MS. Tight junctions: from simple barriers to multifunctional molecular gates. Nat Rev Mol Cell Biol. (2016) 17:564–80. doi: 10.1038/nrm.2016.80, PMID: 27353478

[88] Gill SR Pop M Deboy RT Eckburg PB Turnbaugh PJ Samuel BS. Metagenomic analysis of the human distal gut microbiome. Science. (2006) 312:1355–9. doi: 10.1126/science.1124234, PMID: 16741115 PMC3027896

[89] Ng KM Ferreyra JA Higginbottom SK Lynch JB Kashyap PC Gopinath S. Microbiota-liberated host sugars facilitate post-antibiotic expansion of enteric pathogens. Nature. (2013) 502:96–9. doi: 10.1038/nature12503, PMID: 23995682 PMC3825626

[90] Quigley EMM. Gut microbiome as a clinical tool in gastrointestinal disease management: are we there yet? Nat Rev Gastroenterol Hepatol. (2017) 14:315–20. doi: 10.1038/nrgastro.2017.29, PMID: 28356581

[91] Eckburg PB Bik EM Bernstein CN Purdom E Dethlefsen L Sargent M. Diversity of the human intestinal microbial flora. Science. (2005) 308:1635–8. doi: 10.1126/science.1110591, PMID: 15831718 PMC1395357

[92] Thursby E Juge N. Introduction to the human gut microbiota. Biochem J. (2017) 474:1823–36. doi: 10.1042/BCJ20160510, PMID: 28512250 PMC5433529

[93] Bajaj JS. Alcohol, liver disease and the gut microbiota. Nat Rev Gastroenterol Hepatol. (2019) 16:235–46. doi: 10.1038/s41575-018-0099-1, PMID: 30643227

[94] Belkaid Y Hand TW. Role of the microbiota in immunity and inflammation. Cell. (2014) 157:121–41. doi: 10.1016/j.cell.2014.03.011, PMID: 24679531 PMC4056765

[95] Hou K Wu ZX Chen XY Wang JQ Zhang D Xiao C. Microbiota in health and diseases. Signal Transduct Target Ther. (2022) 7:135. doi: 10.1038/s41392-022-00974-4, PMID: 35461318 PMC9034083

[96] Vancamelbeke M Vermeire S. The intestinal barrier: a fundamental role in health and disease. Expert Rev Gastroenterol Hepatol. (2017) 11:821–34. doi: 10.1080/17474124.2017.1343143, PMID: 28650209 PMC6104804

[97] Brun P Castagliuolo I Di Leo V Buda A Pinzani M Palu G. Increased intestinal permeability in obese mice: new evidence in the pathogenesis of non-alcoholic steatohepatitis. Am J Physiol Gastrointest Liver Physiol. (2007) 292:G518–25. doi: 10.1152/ajpgi.00024.2006, PMID: 17023554

[98] Cui Y Wang Q Chang R Zhou X Xu C. Intestinal barrier function-non-alcoholic fatty liver disease interactions and possible role of gut microbiota. J Agric Food Chem. (2019) 67:2754–62. doi: 10.1021/acs.jafc.9b00080, PMID: 30798598

[99] Groschwitz KR Hogan SP. Intestinal barrier function: molecular regulation and disease pathogenesis. J Allergy Clin Immunol. (2009) 124:3–20. doi: 10.1016/j.jaci.2009.05.038 19560575 PMC4266989

[100] Han R Qiu H Zhong J Zheng N Li B Hong Y. Si Miao formula attenuates non-alcoholic fatty liver disease by modulating hepatic lipid metabolism and gut microbiota. Phytomedicine. (2021) 85:153544. doi: 10.1016/j.phymed.2021.153544, PMID: 33773192

[101] Liu L Fu Q Li T Shao K Zhu X Cong Y. Gut microbiota and butyrate contribute to non-alcoholic fatty liver disease in premenopause due to estrogen deficiency. PLoS One. (2022) 17:e0262855. doi: 10.1371/journal.pone.0262855, PMID: 35108315 PMC8809533

[102] Liu X Sun R Li Z Xiao R Lv P Sun X. Luteolin alleviates non-alcoholic fatty liver disease in rats via restoration of intestinal mucosal barrier damage and microbiota imbalance involving in gut-liver axis. Arch Biochem Biophys. (2021) 711:109019. doi: 10.1016/j.abb.2021.109019 34478730

[103] Miele L Valenza V La Torre G Montalto M Cammarota G Ricci R. Increased intestinal permeability and tight junction alterations in non-alcoholic fatty liver disease. Hepatology. (2009) 49:1877–87. doi: 10.1002/hep.22848 19291785

[104] Parlesak A Schafer C Schutz T Bode JC Bode C. Increased intestinal permeability to macromolecules and endotoxemia in patients with chronic alcohol abuse in different stages of alcohol-induced liver disease. J Hepatol. (2000) 32:742–7. doi: 10.1016/S0168-8278(00)80242-1, PMID: 10845660

[105] Rao RK Seth A Sheth P. Recent advances in alcoholic liver disease I. Role of intestinal permeability and endotoxemia in alcoholic liver disease. Am J Physiol Gastrointest Liver Physiol. (2004) 286:G881–4. doi: 10.1152/ajpgi.00006.2004, PMID: 15132946

[106] Tranah TH Vijay GK Ryan JM Shawcross DL. Systemic inflammation and ammonia in hepatic encephalopathy. Metab Brain Dis. (2013) 28:1–5. doi: 10.1007/s11011-012-9370-2, PMID: 23224356

[107] Xu Y Huang X Huangfu B Hu Y Xu J Gao R. Sulforaphane ameliorates non-alcoholic fatty liver disease induced by high-fat and high-fructose diet via LPS/TLR4 in the gut-liver Axis. Nutrients. (2023) 15:743. doi: 10.3390/nu15030743, PMID: 36771448 PMC9920698

[108] Xue J Zhao M Liu Y Jia X Zhang X Gu Q. Hydrogen inhalation ameliorates hepatic inflammation and modulates gut microbiota in rats with high-fat diet-induced non-alcoholic fatty liver disease. Eur J Pharmacol. (2023) 947:175698. doi: 10.1016/j.ejphar.2023.175698, PMID: 36997047

[109] Yang T Yang H Heng C Wang H Chen S Hu Y. Amelioration of non-alcoholic fatty liver disease by sodium butyrate is linked to the modulation of intestinal tight junctions in db/db mice. Food Funct. (2020) 11:10675–89. doi: 10.1039/D0FO01954B, PMID: 33216087

[110] Elamin E Masclee A Troost F Pieters HJ Keszthelyi D Aleksa K. Ethanol impairs intestinal barrier function in humans through mitogen activated protein kinase signaling: a combined in vivo and in vitro approach. PLoS One. (2014) 9:e107421. doi: 10.1371/journal.pone.0107421, PMID: 25226407 PMC4165763

[111] Li X Wang C Nie J Lv D Wang T Xu Y. Toll-like receptor 4 increases intestinal permeability through up-regulation of membrane PKC activity in alcoholic steatohepatitis. Alcohol. (2013) 47:459–65. doi: 10.1016/j.alcohol.2013.05.004, PMID: 23871536

[112] Rao RK. Acetaldehyde-induced increase in paracellular permeability in Caco-2 cell monolayer. Alcohol Clin Exp Res. (1998) 22:1724–30. doi: 10.1111/j.1530-0277.1998.tb03972.x, PMID: 9835287

[113] Rao RK. Acetaldehyde-induced barrier disruption and paracellular permeability in Caco-2 cell monolayer. Methods Mol Biol. (2008) 447:171–83. doi: 10.1007/978-1-59745-242-7_13, PMID: 18369919

[114] Zhong W Li Q Zhang W Sun Q Sun X Zhou Z. Modulation of intestinal barrier and bacterial endotoxin production contributes to the beneficial effect of nicotinic acid on alcohol-induced endotoxemia and hepatic inflammation in rats. Biomol Ther. (2015) 5:2643–58. doi: 10.3390/biom5042643, PMID: 26501337 PMC4693251

[115] Zhong W Mcclain CJ Cave M Kang YJ Zhou Z. The role of zinc deficiency in alcohol-induced intestinal barrier dysfunction. Am J Physiol Gastrointest Liver Physiol. (2010) 298:G625–33. doi: 10.1152/ajpgi.00350.2009, PMID: 20167873 PMC2867425

[116] Atkinson KJ Rao RK. Role of protein tyrosine phosphorylation in acetaldehyde-induced disruption of epithelial tight junctions. Am J Physiol Gastrointest Liver Physiol. (2001) 280:G1280–8. doi: 10.1152/ajpgi.2001.280.6.G1280, PMID: 11352822

[117] Dunagan M Chaudhry K Samak G Rao RK. Acetaldehyde disrupts tight junctions in Caco-2 cell monolayers by a protein phosphatase 2A-dependent mechanism. Am J Physiol Gastrointest Liver Physiol. (2012) 303:G1356–64. doi: 10.1152/ajpgi.00526.2011 23064762 PMC4073985

[118] Sheth P Seth A Atkinson KJ Gheyi T Kale G Giorgianni F. Acetaldehyde dissociates the PTP1B-E-cadherin-beta-catenin complex in Caco-2 cell monolayers by a phosphorylation-dependent mechanism. Biochem J. (2007) 402:291–300. doi: 10.1042/BJ20060665, PMID: 17087658 PMC1798442

[119] Li Y Liu T Yan C Xie R Guo Z Wang S. Diammonium glycyrrhizinate protects against non-alcoholic fatty liver disease in mice through modulation of gut microbiota and restoration of intestinal barrier. Mol Pharm. (2018) 15:3860–70. doi: 10.1021/acs.molpharmaceut.8b00347, PMID: 30036479

[120] Kaushal K Agarwal S Sharma S Goswami P Singh N Sachdev V. Demonstration of gut-barrier dysfunction in early stages of non-alcoholic fatty liver disease: a proof-of-concept study. J Clin Exp Hepatol. (2022) 12:1102–13. doi: 10.1016/j.jceh.2022.01.006, PMID: 35814507 PMC9257921

[121] Dubinkina VB Tyakht AV Odintsova VY Yarygin KS Kovarsky BA Pavlenko AV. Links of gut microbiota composition with alcohol dependence syndrome and alcoholic liver disease. Microbiome. (2017) 5:141. doi: 10.1186/s40168-017-0359-2, PMID: 29041989 PMC5645934

[122] Hartmann P Seebauer CT Schnabl B. Alcoholic liver disease: the gut microbiome and liver cross talk. Alcohol Clin Exp Res. (2015) 39:763–75. doi: 10.1111/acer.12704, PMID: 25872593 PMC4402724

[123] Weiss GA Hennet T. Mechanisms and consequences of intestinal dysbiosis. Cell Mol Life Sci. (2017) 74:2959–77. doi: 10.1007/s00018-017-2509-x, PMID: 28352996 PMC11107543

[124] Vassallo G Mirijello A Ferrulli A Antonelli M Landolfi R Gasbarrini A. Review article: alcohol and gut microbiota – the possible role of gut microbiota modulation in the treatment of alcoholic liver disease. Aliment Pharmacol Ther. (2015) 41:917–27. doi: 10.1111/apt.13164, PMID: 25809237

[125] Boltin D Niv Y. Pharmacological and alimentary alteration of the gastric barrier. Best Pract Res Clin Gastroenterol. (2014) 28:981–94. doi: 10.1016/j.bpg.2014.09.001, PMID: 25439065

[126] Franke A Teyssen S Singer MV. Alcohol-related diseases of the esophagus and stomach. Dig Dis. (2005) 23:204–13. doi: 10.1159/000090167, PMID: 16508284

[127] Teyssen S Gonzalez-Calero G Schimiczek M Singer MV. Maleic acid and succinic acid in fermented alcoholic beverages are the stimulants of gastric acid secretion. J Clin Invest. (1999) 103:707–13. doi: 10.1172/JCI3620 10074488 PMC408116

[128] Brenner H Rothenbacher D Bode G Adler G. Relation of smoking and alcohol and coffee consumption to active *Helicobacter pylori* infection: cross sectional study. BMJ. (1997) 315:1489–92. doi: 10.1136/bmj.315.7121.1489, PMID: 9420488 PMC2127930

[129] Capurso G Lahner E. The interaction between smoking, alcohol and the gut microbiome. Best Pract Res Clin Gastroenterol. (2017) 31:579–88. doi: 10.1016/j.bpg.2017.10.006, PMID: 29195678

[130] Yan AW Fouts DE Brandl J Starkel P Torralba M Schott E. Enteric dysbiosis associated with a mouse model of alcoholic liver disease. Hepatology. (2011) 53:96–105. doi: 10.1002/hep.24018, PMID: 21254165 PMC3059122

[131] Chen G Shi F Yin W Guo Y Liu A Shuai J. Gut microbiota dysbiosis: the potential mechanisms by which alcohol disrupts gut and brain functions. Front Microbiol. (2022) 13:916765. doi: 10.3389/fmicb.2022.916765, PMID: 35966709 PMC9372561

[132] Wang Y Kirpich I Liu Y Ma Z Barve S Mcclain CJ. *Lactobacillus rhamnosus* GG treatment potentiates intestinal hypoxia-inducible factor, promotes intestinal integrity and ameliorates alcohol-induced liver injury. Am J Pathol. (2011) 179:2866–75. doi: 10.1016/j.ajpath.2011.08.039, PMID: 22093263 PMC3260853

[133] Wang Y Liu Y Sidhu A Ma Z Mcclain C Feng W. *Lactobacillus rhamnosus* GG culture supernatant ameliorates acute alcohol-induced intestinal permeability and liver injury. Am J Physiol Gastrointest Liver Physiol. (2012) 303:G32–41. doi: 10.1152/ajpgi.00024.2012, PMID: 22538402 PMC3404581

[134] Zhao H Zhao C Dong Y Zhang M Wang Y Li F. Inhibition of miR122a by *Lactobacillus rhamnosus* GG culture supernatant increases intestinal occludin expression and protects mice from alcoholic liver disease. Toxicol Lett. (2015) 234:194–200. doi: 10.1016/j.toxlet.2015.03.002, PMID: 25746479

[135] Meroni M Longo M Dongiovanni P. Alcohol or gut microbiota: who is the guilty? Int J Mol Sci. (2019) 20:4568. doi: 10.3390/ijms20184568, PMID: 31540133 PMC6770333

[136] Philips CA Pande A Shasthry SM Jamwal KD Khillan V Chandel SS. Healthy donor fecal microbiota transplantation in steroid-ineligible severe alcoholic hepatitis: a pilot study. Clin Gastroenterol Hepatol. (2017) 15:600–2. doi: 10.1016/j.cgh.2016.10.029, PMID: 27816755

[137] Bjorkhaug ST Aanes H Neupane SP Bramness JG Malvik S Henriksen C. Characterization of gut microbiota composition and functions in patients with chronic alcohol overconsumption. Gut Microbes. (2019) 10:663–75. doi: 10.1080/19490976.2019.1580097, PMID: 30894059 PMC6866679

[138] Bull-Otterson L Feng W Kirpich I Wang Y Qin X Liu Y. Metagenomic analyses of alcohol induced pathogenic alterations in the intestinal microbiome and the effect of *Lactobacillus rhamnosus* GG treatment. PLoS One. (2013) 8:e53028. doi: 10.1371/journal.pone.0053028, PMID: 23326376 PMC3541399

[139] Hartmann P Chen WC Schnabl B. The intestinal microbiome and the leaky gut as therapeutic targets in alcoholic liver disease. Front Physiol. (2012) 3:402. doi: 10.3389/fphys.2012.00402, PMID: 23087650 PMC3468817

[140] Lang S Fairfied B Gao B Duan Y Zhang X Fouts DE. Changes in the fecal bacterial microbiota associated with disease severity in alcoholic hepatitis patients. Gut Microbes. (2020) 12:1785251. doi: 10.1080/19490976.2020.1785251, PMID: 32684075 PMC7524371

[141] Lin D Jiang X Zhao Y Zhai X Yang X. Komagataeibacter hansenii CGMCC 3917 alleviates alcohol-induced liver injury by regulating fatty acid metabolism and intestinal microbiota diversity in mice. Food Funct. (2020) 11:4591–604. doi: 10.1039/C9FO02040C, PMID: 32432239

[142] Ming L Qiao X Yi L Siren D He J Hai L. Camel milk modulates ethanol-induced changes in the gut microbiome and transcriptome in a mouse model of acute alcoholic liver disease. J Dairy Sci. (2020) 103:3937–49. doi: 10.3168/jds.2019-17247, PMID: 32171514

[143] Mutlu E Keshavarzian A Engen P Forsyth CB Sikaroodi M Gillevet P. Intestinal dysbiosis: a possible mechanism of alcohol-induced endotoxemia and alcoholic steatohepatitis in rats. Alcohol Clin Exp Res. (2009) 33:1836–46. doi: 10.1111/j.1530-0277.2009.01022.x, PMID: 19645728 PMC3684271

[144] Mutlu EA Gillevet PM Rangwala H Sikaroodi M Naqvi A Engen PA. Colonic microbiome is altered in alcoholism. Am J Physiol Gastrointest Liver Physiol. (2012) 302:G966–78. doi: 10.1152/ajpgi.00380.2011, PMID: 22241860 PMC3362077

[145] Cresci GA Bush K Nagy LE. Tributyrin supplementation protects mice from acute ethanol-induced gut injury. Alcohol Clin Exp Res. (2014) 38:1489–501. doi: 10.1111/acer.12428, PMID: 24890666 PMC4185400

[146] Lee JE Ha JS Park HY Lee E. Alteration of gut microbiota composition by short-term low-dose alcohol intake is restored by fermented rice liquor in mice. Food Res Int. (2020) 128:108800. doi: 10.1016/j.foodres.2019.108800, PMID: 31955762

[147] Kobayashi T Iwaki M Nakajima A Nogami A Yoneda M. Current research on the pathogenesis of NAFLD/NASH and the gut-liver axis: gut microbiota, dysbiosis, and leaky-gut syndrome. Int J Mol Sci. (2022) 23:1689. doi: 10.3390/ijms231911689, PMID: 36232990 PMC9570241

[148] Rau M Rehman A Dittrich M Groen AK Hermanns HM Seyfried F. Fecal SCFAs and SCFA-producing bacteria in gut microbiome of human NAFLD as a putative link to systemic T-cell activation and advanced disease. United European Gastroenterol J. (2018) 6:1496–507. doi: 10.1177/2050640618804444, PMID: 30574320 PMC6297934

[149] Boursier J Mueller O Barret M Machado M Fizanne L Araujo-Perez F. The severity of non-alcoholic fatty liver disease is associated with gut dysbiosis and shift in the metabolic function of the gut microbiota. Hepatology. (2016) 63:764–75. doi: 10.1002/hep.28356, PMID: 26600078 PMC4975935

[150] Zhang R Yan Z Zhong H Luo R Liu W Xiong S. Gut microbial metabolites in MASLD: implications of mitochondrial dysfunction in the pathogenesis and treatment. Hepatol Commun. (2024) 8:484. doi: 10.1097/HC9.0000000000000484, PMID: 38967596 PMC11227362

[151] Monga Kravetz A Testerman T Galuppo B Graf J Pierpont B Siebel S. Effect of gut microbiota and PNPLA3 rs738409 variant on non-alcoholic fatty liver disease (NAFLD) in obese youth. J Clin Endocrinol Metab. (2020) 105:e3575–85. doi: 10.1210/clinem/dgaa382, PMID: 32561908 PMC7458486

[152] Zhuge A Li S Lou P Wu W Wang K Yuan Y. Longitudinal 16S rRNA sequencing reveals relationships among alterations of gut microbiota and non-alcoholic fatty liver disease progression in mice. Microbiol Spectr. (2022) 10:e0004722. doi: 10.1128/spectrum.00047-22, PMID: 35647690 PMC9241867

[153] Yuan J Chen C Cui J Lu J Yan C Wei X. Fatty liver disease caused by high-alcohol-producing *Klebsiella pneumoniae*. Cell Metab. (2019) 30:e677. doi: 10.1016/j.cmet.2019.11.006, PMID: 31801057

[154] Ferro D Baratta F Pastori D Cocomello N Colantoni A Angelico F. New insights into the pathogenesis of non-alcoholic fatty liver disease: gut-derived lipopolysaccharides and oxidative stress. Nutrients. (2020) 12:2762. doi: 10.3390/nu12092762, PMID: 32927776 PMC7551294

[155] Kirpich IA Marsano LS Mcclain CJ. Gut-liver axis, nutrition, and non-alcoholic fatty liver disease. Clin Biochem. (2015) 48:923–30. doi: 10.1016/j.clinbiochem.2015.06.023, PMID: 26151226 PMC4558208

[156] Poeta M Pierri L Vajro P. Gut-liver Axis derangement in non-alcoholic fatty liver disease. Children. (2017) 4:66. doi: 10.3390/children4080066, PMID: 28767077 PMC5575588

[157] Dobrijevic D Pastor K Nastic N Ozogul F Krulj J Kokic B. Betaine as a functional ingredient: metabolism, health-promoting attributes, food sources, applications and analysis methods. Molecules. (2023) 28:4824. doi: 10.3390/molecules28124824, PMID: 37375378 PMC10302777

[158] Ghosh S Whitley CS Haribabu B Jala VR. Regulation of intestinal barrier function by microbial metabolites. Cell Mol Gastroenterol Hepatol. (2021) 11:1463–82. doi: 10.1016/j.jcmgh.2021.02.007, PMID: 33610769 PMC8025057

[159] Kim DH Sung B Kang YJ Jang JY Hwang SY Lee Y. Anti-inflammatory effects of betaine on AOM/DSS-induced colon tumorigenesis in ICR male mice. Int J Oncol. (2014) 45:1250–6. doi: 10.3892/ijo.2014.2515, PMID: 24969167

[160] Day CR Kempson SA. Betaine chemistry, roles, and potential use in liver disease. Biochim Biophys Acta. (2016) 1860:1098–106. doi: 10.1016/j.bbagen.2016.02.001, PMID: 26850693

[161] Sakamoto A Ono H Mizoguchi N Sakura N. Betaine and homocysteine concentrations in infant formulae and breast milk. Pediatr Int. (2001) 43:637–40. doi: 10.1046/j.1442-200X.2001.01465.x, PMID: 11737741

[162] Zhao N Yang S Jia Y Sun B He B Zhao R. Maternal betaine supplementation attenuates glucocorticoid-induced hepatic lipid accumulation through epigenetic modification in adult offspring rats. J Nutr Biochem. (2018) 54:105–12. doi: 10.1016/j.jnutbio.2017.12.003, PMID: 29331496

[163] Willingham BD Ragland TJ Ormsbee MJ. Betaine supplementation may improve heat tolerance: potential mechanisms in humans. Nutrients. (2020) 12:2939. doi: 10.3390/nu12102939, PMID: 32992781 PMC7599524

[164] Zhao G He F Wu C Li P Li N Deng J. Betaine in inflammation: mechanistic aspects and applications. Front Immunol. (2018) 9:1070. doi: 10.3389/fimmu.2018.01070, PMID: 29881379 PMC5976740

[165] Ross AB Zangger A Guiraud SP. Cereal foods are the major source of betaine in the Western diet--analysis of betaine and free choline in cereal foods and updated assessments of betaine intake. Food Chem. (2014) 145:859–65. doi: 10.1016/j.foodchem.2013.08.122, PMID: 24128557

[166] Altinisik S Zeidan H Yilmaz MD Marti ME. Reactive extraction of betaine from Sugar beet processing byproducts. ACS Omega. (2023) 8:11029–38. doi: 10.1021/acsomega.2c07845, PMID: 37008146 PMC10061657

[167] Sookoian S Puri P Castano GO Scian R Mirshahi F Sanyal AJ. Non-alcoholic steatohepatitis is associated with a state of betaine-insufficiency. Liver Int. (2017) 37:611–9. doi: 10.1111/liv.13249, PMID: 27614103

[168] Barak AJ Baker H Tuma DJ. Influence of ethanol on in-vivo levels of hepatic Methylators betaine and N5-Methyltetrahydrofolate in the rat. IRCS Med Sci. (1981) 9:527–8.

[169] Barak AJ Beckenhauer HC Tuma DJ Badakhsh S. Effects of prolonged ethanol feeding on methionine metabolism in rat liver. Biochem Cell Biol. (1987) 65:230–3. doi: 10.1139/o87-029, PMID: 3580171

[170] Ganesan B Anandan R Lakshmanan PT. Studies on the protective effects of betaine against oxidative damage during experimentally induced restraint stress in Wistar albino rats. Cell Stress Chaperones. (2011) 16:641–52. doi: 10.1007/s12192-011-0276-4, PMID: 21717086 PMC3220389

[171] Hayes KC Pronczuk A Cook MW Robbins MC. Betaine in sub-acute and sub-chronic rat studies. Food Chem Toxicol. (2003) 41:1685–700. doi: 10.1016/S0278-6915(03)00196-0, PMID: 14563394

[172] Ducker GS Rabinowitz JD. One-carbon metabolism in health and disease. Cell Metab. (2017) 25:27–42. doi: 10.1016/j.cmet.2016.08.009, PMID: 27641100 PMC5353360

[173] Ratriyanto A Mosenthin R. Osmoregulatory function of betaine in alleviating heat stress in poultry. J Anim Physiol Anim Nutr. (2018) 102:1634–50. doi: 10.1111/jpn.12990 30238641

[174] Ueland PM. Choline and betaine in health and disease. J Inherit Metab Dis. (2011) 34:3–15. doi: 10.1007/s10545-010-9088-4, PMID: 20446114

[175] Zeisel SH Blusztajn JK. Choline and human nutrition. Annu Rev Nutr. (1994) 14:269–96. doi: 10.1146/annurev.nu.14.070194.001413 7946521

[176] Teixido N Canamas TP Usall J Torres R Magan N Vinas I. Accumulation of the compatible solutes, glycine-betaine and ectoine, in osmotic stress adaptation and heat shock cross-protection in the biocontrol agent *Pantoea agglomerans* CPA-2. Lett Appl Microbiol. (2005) 41:248–52. doi: 10.1111/j.1472-765X.2005.01757.x, PMID: 16108915

[177] Wang Z Yao T Pini M Zhou Z Fantuzzi G Song Z. Betaine improved adipose tissue function in mice fed a high-fat diet: a mechanism for hepatoprotective effect of betaine in non-alcoholic fatty liver disease. Am J Physiol Gastrointest Liver Physiol. (2010) 298:G634–42. doi: 10.1152/ajpgi.00249.2009, PMID: 20203061 PMC2867421

[178] Ismaeel A. Effects of betaine supplementation on muscle strength and power: a systematic review. J Strength Cond Res. (2017) 31:2338–46. doi: 10.1519/JSC.0000000000001959, PMID: 28426517

[179] Feng Q Kalari K Fridley BL Jenkins G Ji Y Abo R. Betaine-homocysteine methyltransferase: human liver genotype-phenotype correlation. Mol Genet Metab. (2011) 102:126–33. doi: 10.1016/j.ymgme.2010.10.010, PMID: 21093336 PMC3053054

[180] Li F Feng Q Lee C Wang S Pelleymounter LL Moon I. Human betaine-homocysteine methyltransferase (BHMT) and BHMT2: common gene sequence variation and functional characterization. Mol Genet Metab. (2008) 94:326–35. doi: 10.1016/j.ymgme.2008.03.013, PMID: 18457970 PMC2515933

[181] Pellanda H. Betaine homocysteine methyltransferase (BHMT)-dependent remethylation pathway in human healthy and tumoral liver. Clin Chem Lab Med. (2013) 51:617–21. doi: 10.1515/cclm-2012-0689, PMID: 23449526

[182] Pellanda H Namour F Fofou-Caillierez M Bressenot A Alberto JM Chery C. A splicing variant leads to complete loss of function of betaine-homocysteine methyltransferase (BHMT) gene in hepatocellular carcinoma. Int J Biochem Cell Biol. (2012) 44:385–92. doi: 10.1016/j.biocel.2011.11.014, PMID: 22138536

[183] Barak AJ Beckenhauer HC Kharbanda KK Tuma DJ. Chronic ethanol consumption increases homocysteine accumulation in hepatocytes. Alcohol. (2001) 25:77–81. doi: 10.1016/S0741-8329(01)00168-9, PMID: 11747976

[184] Kharbanda KK. Alcoholic liver disease and methionine metabolism. Semin Liver Dis. (2009) 29:155–65. doi: 10.1055/s-0029-1214371 19387915

[185] Kharbanda KK Todero SL Ward BW Cannella JJ Tuma DJ. Betaine administration corrects ethanol-induced defective VLDL secretion. Mol Cell Biochem. (2009) 327:75–8. doi: 10.1007/s11010-009-0044-2, PMID: 19219625

[186] Osna NA White RL Donohue TM Jr Beard MR Tuma DJ Kharbanda KK. Impaired methylation as a novel mechanism for proteasome suppression in liver cells. Biochem Biophys Res Commun. (2010) 391:1291–6. doi: 10.1016/j.bbrc.2009.12.074, PMID: 20026058 PMC2812660

[187] Jung YS Kim SJ Kwon Do Y Ahn CW Kim YS Choi DW. Alleviation of alcoholic liver injury by betaine involves an enhancement of antioxidant defense via regulation of sulfur amino acid metabolism. Food Chem Toxicol. (2013) 62:292–8. doi: 10.1016/j.fct.2013.08.049, PMID: 23994088

[188] Shi QZ Wang LW Zhang W Gong ZJ. Betaine inhibits toll-like receptor 4 expression in rats with ethanol-induced liver injury. World J Gastroenterol. (2010) 16:897–903. doi: 10.3748/wjg.v16.i7.897 PMID: 20143470 PMC2825338

[189] Ji C Kaplowitz N. Betaine decreases hyperhomocysteinemia, endoplasmic reticulum stress, and liver injury in alcohol-fed mice. Gastroenterology. (2003) 124:1488–99. doi: 10.1016/S0016-5085(03)00276-2, PMID: 12730887

[190] Li J Li XM Caudill M Malysheva O Bardag-Gorce F Oliva J. Betaine feeding prevents the blood alcohol cycle in rats fed alcohol continuously for 1 month using the rat intragastric tube feeding model. Exp Mol Pathol. (2011) 91:540–7. doi: 10.1016/j.yexmp.2011.05.009, PMID: 21708146 PMC3185137

[191] Kathirvel E Morgan K Nandgiri G Sandoval BC Caudill MA Bottiglieri T. Betaine improves non-alcoholic fatty liver and associated hepatic insulin resistance: a potential mechanism for hepatoprotection by betaine. Am J Physiol Gastrointest Liver Physiol. (2010) 299:G1068–77. doi: 10.1152/ajpgi.00249.2010, PMID: 20724529 PMC2993168

[192] Mukherjee S Bernard T Kharbanda K Barak AJ Sorrell MF Tuma DJ. Impact of betaine on hepatic fibrosis and homocysteine in non-alcoholic steatohepatitis-a prospective cohort study. Open Transl. J. (2011) 3:1–4. doi: 10.2174/1876399501103010001

[193] Song Z Deaciuc I Zhou Z Song M Chen T Hill D. Involvement of AMP-activated protein kinase in beneficial effects of betaine on high-sucrose diet-induced hepatic steatosis. Am J Physiol Gastrointest Liver Physiol. (2007) 293:G894–902. doi: 10.1152/ajpgi.00133.2007, PMID: 17702954 PMC4215798

[194] Tokar JL Berg CL. Therapeutic options in non-alcoholic fatty liver disease. Curr Treat Options Gastroenterol. (2002) 5:425–36. doi: 10.1007/s11938-002-0030-1 12408779

[195] Zhang W Wang LW Wang LK Li X Zhang H Luo LP. Betaine protects against high-fat-diet-induced liver injury by inhibition of high-mobility group box 1 and toll-like receptor 4 expression in rats. Dig Dis Sci. (2013) 58:3198–206. doi: 10.1007/s10620-013-2775-x 23861108

[196] Rehman A Mehta KJ. Betaine in ameliorating alcohol-induced hepatic steatosis. Eur J Nutr. (2022) 61:1167–76. doi: 10.1007/s00394-021-02738-2, PMID: 34817678 PMC8921017

[197] Thomes PG Bligh SM Kharbanda KK. Multiple roles of betaine against alcohol-induced liver injury In: Preedy VR, editor. Betaine, chemistry, analysis, function and effects. UK: Royal Society of Chemistry (2015). 285–310.

[198] Cho I Yamanishi S Cox L Methe BA Zavadil J Li K. Antibiotics in early life alter the murine colonic microbiome and adiposity. Nature. (2012) 488:621–6. doi: 10.1038/nature11400, PMID: 22914093 PMC3553221

[199] Kettunen H Tiihonen K Peuranen S Saarinen MT Remus JC. Dietary betaine accumulates in the liver and intestinal tissue and stabilizes the intestinal epithelial structure in healthy and coccidia-infected broiler chicks. Comp Biochem Physiol A Mol Integr Physiol. (2001) 130:759–69. doi: 10.1016/S1095-6433(01)00410-X, PMID: 11691612

[200] Liu W Yuan Y Sun C Balasubramanian B Zhao Z An L. Effects of dietary betaine on growth performance, digestive function, carcass traits, and meat quality in indigenous yellow-feathered broilers under long-term heat stress. Animals. (2019) 9:506. doi: 10.3390/ani9080506 31370305 PMC6720770

[201] Yang Z Yang JJ Zhu PJ Han HM Wan XL Yang HM. Effects of betaine on growth performance, intestinal health, and immune response of goslings challenged with lipopolysaccharide. Poult Sci. (2022) 101:102153. doi: 10.1016/j.psj.2022.102153, PMID: 36179650 PMC9523388

[202] Wu J He C Bu J Luo Y Yang S Ye C. Betaine attenuates LPS-induced downregulation of Occludin and Claudin-1 and restores intestinal barrier function. BMC Vet Res. (2020) 16:75. doi: 10.1186/s12917-020-02298-3, PMID: 32131830 PMC7057534

[203] Chen Q Wang Y Jiao F Shi C Pei M Wang L. Betaine inhibits toll-like receptor 4 responses and restores intestinal microbiota in acute liver failure mice. Sci Rep. (2020) 10:21850. doi: 10.1038/s41598-020-78935-6, PMID: 33318565 PMC7736280

[204] Zhao N Yang Y Chen C Jing T Hu Y Xu H. Betaine supplementation alleviates dextran sulfate sodium-induced colitis via regulating the inflammatory response, enhancing the intestinal barrier, and altering gut microbiota. Food Funct. (2022) 13:12814–26. doi: 10.1039/D2FO02942A, PMID: 36422855

[205] Kharbanda KK. Role of transmethylation reactions in alcoholic liver disease. World J Gastroenterol. (2007) 13:4947–54. doi: 10.3748/wjg.v13.i37.4947, PMID: 17854136 PMC4434617

[206] Kharbanda KK Rogers DD Mailliard ME Siford GL Barak AJ Beckenhauer HC. A comparison of the effects of betaine and S-adenosylmethionine on ethanol-induced changes in methionine metabolism and steatosis in rat hepatocytes. J Nutr. (2005) 135:519–24. doi: 10.1093/jn/135.3.519, PMID: 15735087

[207] Kharbanda KK Ronis MJJ Shearn CT Petersen DR Zakhari S Warner DR. Role of nutrition in alcoholic liver disease: summary of the symposium at the ESBRA 2017 congress. Biomol Ther. (2018) 8:8. doi: 10.3390/biom8020016, PMID: 29587455 PMC6022870

[208] Kharbanda KK Todero SL King AL Osna NA Mcvicker BL Tuma DJ. Betaine treatment attenuates chronic ethanol-induced hepatic steatosis and alterations to the mitochondrial respiratory chain proteome. Int J Hepatol. (2012) 2012:962183:1–10. doi: 10.1155/2012/962183, PMID: 22187660 PMC3235488

[209] Mukherjee S. Betaine and non-alcoholic steatohepatitis: back to the future? World J Gastroenterol. (2011) 17:3663–4. doi: 10.3748/wjg.v17.i32.3663, PMID: 21990946 PMC3181450

[210] Dou X Xia Y Chen J Qian Y Li S Zhang X. Rectification of impaired adipose tissue methylation status and lipolytic response contributes to hepatoprotective effect of betaine in a mouse model of alcoholic liver disease. Br J Pharmacol. (2014) 171:4073–86. doi: 10.1111/bph.12765, PMID: 24819676 PMC4243980

[211] Chopyk DM Grakoui A. Contribution of the intestinal microbiome and gut barrier to hepatic disorders. Gastroenterology. (2020) 159:849–63. doi: 10.1053/j.gastro.2020.04.077, PMID: 32569766 PMC7502510

[212] Alvarenga L Ferreira MS Kemp JA Mafra D. The role of betaine in patients with chronic kidney disease: a narrative review. Curr Nutr Rep. (2022) 11:395–406. doi: 10.1007/s13668-022-00426-z, PMID: 35792998

[213] Ferenc K Sokal-Dembowska A Helma K Motyka E Jarmakiewicz-Czaja S Filip R. Modulation of the gut microbiota by nutrition and its relationship to epigenetics. Int J Mol Sci. (2024) 25:1228. doi: 10.3390/ijms25021228, PMID: 38279228 PMC10816208

[214] Li S Xu S Zhao Y Wang H Feng J. Dietary betaine addition promotes hepatic cholesterol synthesis, bile acid conversion, and export in rats. Nutrients. (2020) 12:1399. doi: 10.3390/nu12051399, PMID: 32414094 PMC7284822

[215] Zhao N Yang S Feng Y Sun B Zhao R. Enhanced hepatic cholesterol accumulation induced by maternal betaine exposure is associated with hypermethylation of CYP7A1 gene promoter. Endocrine. (2019) 64:544–51. doi: 10.1007/s12020-019-01906-z, PMID: 30924082

[216] Li Z Pu J Chen X Chen Y Peng X Cai J. Betaine addition to the diet alleviates intestinal injury in growing rabbits during the summer heat through the AAT/mTOR pathway. J Anim Sci Biotechnol. (2024) 15:41. doi: 10.1186/s40104-024-00998-6, PMID: 38454493 PMC10921597

[217] Chen X Li Z Pu J Cai J Zhao H Jia G. Dietary betaine improves the intestinal health and growth performance of heat-stressed growing rabbits in summer. J Anim Sci. (2023) 101:363. doi: 10.1093/jas/skad363, PMID: 37875147 PMC10684048

[218] Hemarajata P Versalovic J. Effects of probiotics on gut microbiota: mechanisms of intestinal immunomodulation and neuromodulation. Ther Adv Gastroenterol. (2013) 6:39–51. doi: 10.1177/1756283X12459294, PMID: 23320049 PMC3539293

[219] Maftei NM Raileanu CR Balta AA Ambrose L Boev M Marin DB. The potential impact of probiotics on human health: An update on their health-promoting properties. Microorganisms. (2024) 12:234. doi: 10.3390/microorganisms12020234 38399637 PMC10891645

[220] Thomas CM Versalovic J. Probiotics-host communication: modulation of signaling pathways in the intestine. Gut Microbes. (2010) 1:148–63. doi: 10.4161/gmic.1.3.11712, PMID: 20672012 PMC2909492

[221] Cani PD Delzenne NM. The role of the gut microbiota in energy metabolism and metabolic disease. Curr Pharm Des. (2009) 15:1546–58. doi: 10.2174/138161209788168164, PMID: 19442172

[222] Choi MY Fitzpatrick RD Buhler K Mahler M Fritzler MJ. A review and meta-analysis of anti-ribosomal P autoantibodies in systemic lupus erythematosus. Autoimmun Rev. (2020) 19:102463. doi: 10.1016/j.autrev.2020.102463 31927088

[223] Hajirezaee S Ramezani S Ramezani S. Betaine and the probiotic, *Lactobacillus rhamnosus* in the diet of the common carp, *Cyprinus carpio*: effects on growth, digestive enzyme activities, antioxidant system, humoral and mucosal immunity and resistance to *Streptococcus iniae*. Aquacult. Rep. (2024) 38:102282. doi: 10.1016/j.aqrep.2024.102282

[224] Kerksick C Willoughby D. The antioxidant role of glutathione and N-acetyl-cysteine supplements and exercise-induced oxidative stress. J Int Soc Sports Nutr. (2005) 2:38–44. doi: 10.1186/1550-2783-2-2-38, PMID: 18500954 PMC2129149

[225] Sen CK Khanna S Reznick AZ Roy S Packer L. Glutathione regulation of tumor necrosis factor-alpha-induced NF-kappa B activation in skeletal muscle-derived L6 cells. Biochem Biophys Res Commun. (1997) 237:645–9. doi: 10.1006/bbrc.1997.7206, PMID: 9299419

[226] Jewell DE Jackson MI. Dietary betaine interacts with very long chain n-3 polyunsaturated fatty acids to influence fat metabolism and circulating single carbon status in the cat. Animals. (2022) 12:2837. doi: 10.3390/ani12202837 36290222 PMC9597741

[227] Jewell DE Tavener SK Creech R Panickar KS. Betaine and L-carnitine synergistically influence the metabolome and immune response in dogs. Animals. (2024) 14:357. doi: 10.3390/ani14030357, PMID: 38338001 PMC10854714

[228] Lattef FA Khudair KK. Effects of combination of betaine and Kaempferol on induced methionine overload in rats: lipid profile and oxidative stress. Rev. Latinoamericana Hipertensión. (2024) 19:183–90. doi: 10.5281/zenodo.11261767

[229] Lee SY Ko KS. Effects of S-adenosylmethionine and its combinations with taurine and/or betaine on glutathione homeostasis in ethanol-induced acute hepatotoxicity. J Cancer Prev. (2016) 21:164–72. doi: 10.15430/JCP.2016.21.3.164, PMID: 27722142 PMC5051590

[230] Lee SY Ko KS. Protective effects of S-adenosylmethionine and its combinations with taurine and/or betaine against lipopolysaccharide or polyinosinic-polycytidylic acid-induced acute hepatotoxicity. J Cancer Prev. (2016) 21:152–63. doi: 10.15430/JCP.2016.21.3.152, PMID: 27722141 PMC5051589

[231] Li Y Yuan J Sun S Ma F Xiong Y He S. Optimizing growth and antioxidant function in heat-stressed broilers with vitamin C and betaine supplementation. Int J Biometeorol. (2024) 68:1953–60. doi: 10.1007/s00484-024-02717-2, PMID: 38834879

[232] Saleh AA El-Tahan HM Shaban M Morsy WA Genedy S Alzawqari MH. Effect of dietary supplementation of betaine and organic minerals on growth performance, serum biochemical parameters, nutrients digestibility, and growth-related genes in broilers under heat stress. Poult Sci. (2023) 102:103051. doi: 10.1016/j.psj.2023.103051, PMID: 37774520 PMC10550832

[233] Sun L Tan X Liang X Chen H Ou Q Wu Q. Maternal betaine supplementation mitigates maternal high fat diet-induced NAFLD in offspring mice through gut microbiota. Nutrients. (2023) 15:15. doi: 10.3390/nu15020284, PMID: 36678155 PMC9861146

[234] Augustine PC Mcnaughton JL Virtanen E Rosi L. Effect of betaine on the growth performance of chicks inoculated with mixed cultures of avian Eimeria species and on invasion and development of *Eimeria tenella* and *Eimeria acervulina* in vitro and in vivo. Poult Sci. (1997) 76:802–9. doi: 10.1093/ps/76.6.802, PMID: 9181611

[235] Wang W Liu A Chen X Zheng X Fu W Wang G. The potential role of betaine in enhancement of microbial-assisted phytoremediation of benzophenone-3 contaminated soil. Chemosphere. (2022) 307:135783. doi: 10.1016/j.chemosphere.2022.135783, PMID: 35868529

[236] Abu Hafsa SH Centoducati G Hassan AA Maggiolino A Elghandour M Salem AZM. Effects of dietary supplementations of vitamin C, organic selenium, betaine, and pomegranate Peel on alleviating the effect of heat stress on growing rabbits. Animals. (2024) 14:950. doi: 10.3390/ani14060950 38540048 PMC10967313

[237] Wahid ZA Pradista LA Prastowo S Ratriyanto A (2024). Betaine Alter SCFA-producing Bacteria population in laying pullet reared in tropical climate. IOP Conference Series: Earth and Environmental Science 1341.

[238] Yang W Huang L Gao J Wen S Tai Y Chen M. Betaine attenuates chronic alcohol-induced fatty liver by broadly regulating hepatic lipid metabolism. Mol Med Rep. (2017) 16:5225–34. doi: 10.3892/mmr.2017.7295, PMID: 28849079 PMC5647077

[239] Balkan J Oztezcan S Kucuk M Cevikbas U Kocak-Toker N Uysal M. The effect of betaine treatment on triglyceride levels and oxidative stress in the liver of ethanol-treated guinea pigs. Exp Toxicol Pathol. (2004) 55:505–9. doi: 10.1078/0940-2993-00347, PMID: 15384256

[240] Kanbak G Arslan OC Dokumacioglu A Kartkaya K Inal ME. Effects of chronic ethanol consumption on brain synaptosomes and protective role of betaine. Neurochem Res. (2008) 33:539–44. doi: 10.1007/s11064-007-9472-0, PMID: 17763942

[241] Oliva J Bardag-Gorce F Tillman B French SW. Protective effect of quercetin, EGCG, catechin and betaine against oxidative stress induced by ethanol in vitro. Exp Mol Pathol. (2011) 90:295–9. doi: 10.1016/j.yexmp.2011.02.006, PMID: 21352821 PMC3113678

[242] Kwon DY Jung YS Kim SJ Park HK Park JH Kim YC. Impaired sulfur-amino acid metabolism and oxidative stress in non-alcoholic fatty liver are alleviated by betaine supplementation in rats. J Nutr. (2009) 139:63–8. doi: 10.3945/jn.108.094771, PMID: 19056644

[243] Du J Shen L Tan Z Zhang P Zhao X Xu Y. Betaine supplementation enhances lipid metabolism and improves insulin resistance in mice fed a high-fat diet. Nutrients. (2018) 10:131. doi: 10.3390/nu10020131 29373534 PMC5852707

[244] Li L Sun L Liang X Ou Q Tan X Li F. Maternal betaine supplementation ameliorates fatty liver disease in offspring mice by inhibiting hepatic NLRP3 inflammasome activation. Nutr Res Pract. (2023) 17:1084–98. doi: 10.4162/nrp.2023.17.6.1084 38053832 PMC10694418

[245] Abu Ahmad N Raizman M Weizmann N Wasek B Arning E Bottiglieri T. Betaine attenuates pathology by stimulating lipid oxidation in liver and regulating phospholipid metabolism in brain of methionine-choline-deficient rats. FASEB J. (2019) 33:9334–49. doi: 10.1096/fj.201802683R, PMID: 31120771

[246] Veskovic M Mladenovic D Milenkovic M Tosic J Borozan S Gopcevic K. Betaine modulates oxidative stress, inflammation, apoptosis, autophagy, and Akt/mTOR signaling in methionine-choline deficiency-induced fatty liver disease. Eur J Pharmacol. (2019) 848:39–48. doi: 10.1016/j.ejphar.2019.01.043, PMID: 30689995

[247] Veskovic M Labudovic-Borovic M Mladenovic D Jadzic J Jorgacevic B Vukicevic D. Effect of betaine supplementation on liver tissue and ultrastructural changes in methionine-choline-deficient diet-induced NAFLD. Microsc Microanal. (2020) 26:997–1006. doi: 10.1017/S1431927620024265, PMID: 32782033

[248] Abdelmalek MF Angulo P Jorgensen RA Sylvestre PB Lindor KD. Betaine, a promising new agent for patients with non-alcoholic steatohepatitis: results of a pilot study. Am J Gastroenterol. (2001) 96:2711–7. doi: 10.1111/j.1572-0241.2001.04129.x, PMID: 11569700

[249] Abdelmalek MF Sanderson SO Angulo P Soldevila-Pico C Liu C Peter J. Betaine for non-alcoholic fatty liver disease: results of a randomized placebo-controlled trial. Hepatology. (2009) 50:1818–26. doi: 10.1002/hep.23239 19824078

[250] Miglio F Rovati LC Santoro A Setnikar I. Efficacy and safety of oral betaine glucuronate in non-alcoholic steatohepatitis. A double-blind, randomized, parallel-group, placebo-controlled prospective clinical study. Arzneimittelforschung. (2000) 50:722–7. PMID: 10994156 10.1055/s-0031-1300279

[251] Steenge GR Verhoef P Katan MB. Betaine supplementation lowers plasma homocysteine in healthy men and women. J Nutr. (2003) 133:1291–5. doi: 10.1093/jn/133.5.1291, PMID: 12730412

[252] Smolin LA Benevenga NJ Berlow S. The use of betaine for the treatment of homocystinuria. J Pediatr. (1981) 99:467–72. doi: 10.1016/S0022-3476(81)80352-6, PMID: 7264811

[253] Kim SK Kim YC. Attenuation of bacterial lipopolysaccharide-induced hepatotoxicity by betaine or taurine in rats. Food Chem Toxicol. (2002) 40:545–9. doi: 10.1016/S0278-6915(01)00102-8, PMID: 11893413

[254] Park SO Kim WK. Effects of betaine on biological functions in meat-type ducks exposed to heat stress. Poult Sci. (2017) 96:1212–8. doi: 10.3382/ps/pew359, PMID: 27702920

[255] Ali BH Al-Qarawi AA Mousa HM. Stress associated with road transportation in desert sheep and goats, and the effect of pretreatment with xylazine or sodium betaine. Res Vet Sci. (2006) 80:343–8. doi: 10.1016/j.rvsc.2005.07.012, PMID: 16181650

[256] Qu Y Zhang K Pu Y Chang L Wang S Tan Y. Betaine supplementation is associated with the resilience in mice after chronic social defeat stress: a role of brain-gut-microbiota axis. J Affect Disord. (2020) 272:66–76. doi: 10.1016/j.jad.2020.03.095, PMID: 32379622

[257] Barak AJ Beckenhauer HC Badakhsh S Tuma DJ. The effect of betaine in reversing alcoholic steatosis. Alcohol Clin Exp Res. (1997) 21:1100–1102. doi: 10.1111/J.1530-0277.1997.TB04259.X 9309323

